# Exploring the multimodal health‐promoting properties of resveratrol: A comprehensive review

**DOI:** 10.1002/fsn3.3933

**Published:** 2024-01-28

**Authors:** Zargham Faisal, Aimen Mazhar, Syeda Ayesha Batool, Noor Akram, Maleeha Hassan, Muhammad Usman Khan, Muhammad Afzaal, Usman Ul Hassan, Yasir Abbas Shah, Derese Tamiru Desta

**Affiliations:** ^1^ Department of Human Nutrition Bahauddin Zakariya University, Faculty of Food Science and Nutrition Multan Pakistan; ^2^ Department of Food Science Government College University Faisalabad Faisalabad Pakistan; ^3^ Department of Food and Nutrition Government College University Faisalabad Faisalabad Pakistan; ^4^ Department of Dietetics and Nutritional Science University of Sialkot Sialkot Pakistan; ^5^ Department of Food Science and Technology Bahauddin Zakariya University, Faculty of Food Science and Nutrition Multan Pakistan; ^6^ National Institute of Food Science and Technology University of Agriculture Faisalabad Faisalabad Pakistan; ^7^ Natural and Medical Science Research Centre University of Nizwa Nizwa Oman; ^8^ School of Nutrition, Food Science and Technology Hawassa University Hawassa Ethiopia

**Keywords:** antioxidant, gut microbiota, nano‐delivery, polyphenol, resveratrol

## Abstract

Resveratrol, a natural polyphenol in various plants, has gained significant attention for its potential health‐promoting properties. It has been demonstrated, after reviewing various clinical and in vitro studies, that resveratrol possesses potent antioxidant potential. Resveratrol demonstrates cellular component protection by directly neutralizing free radicals (FRs) and enhancing the expression of natural antioxidant enzymes, thereby mitigating oxidative damage to proteins, lipids, and nucleic acids. Clinical trials have shown promising results, indicating that resveratrol supplementation can enhance antioxidant defenses and reduce oxidative damage markers in various populations. In addition to its antioxidant effects, resveratrol exhibits potent anti‐inflammatory properties. It can modulate key inflammatory pathways, such as nuclear factor‐kappa B (NF‐κB) and mitogen‐activated protein kinases (MAPKs), thereby suppressing the production of pro‐inflammatory cytokines and chemokines. Furthermore, resveratrol's multimodal effects extend beyond its antioxidant and anti‐inflammatory properties. It has been discovered to exert regulatory effects on various cellular processes, including apoptosis, cell cycle progression, angiogenesis, and immunological responses. The primary aim of this review paper is to provide a thorough overview of the current knowledge on resveratrol, including its chemical composition, bioaccessibility, clinical effectiveness, and utilization in nanotechnology to enhance its bioavailability. From future perspectives, revising the administration methods for certain contexts and understanding the underlying systems responsible for resveratrol's effects will require further inquiry. For the highest potential health results, advanced trial‐based research is necessary for combinational nano‐delivery of resveratrol.

## Graphical abstract

This review article presents a comprehensive summary of the present understanding of resveratrol, encompassing its chemical composition, bioaccessibility, and clinical efficacy. Additionally, this review discusses the employment of nanotechnology to enhance the bioavailability of resveratrol, to counter different disease conditions.

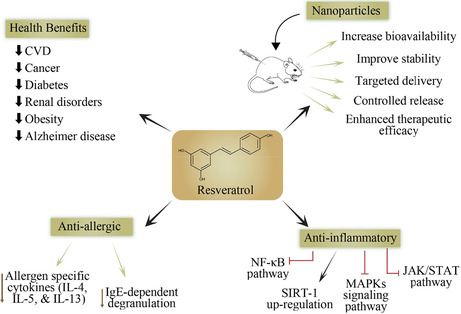

## 1 INTRODUCTION

Resveratrol (RESV) (3,5,4′‐trans‐trihydroxystilbene) is a naturally occurring bioactive compound that belongs to the stilbene family, basically a dietary plant substance that is mostly found in the skin and seeds of grapes, although it has been found in various kinds of plant foods, particularly in the tea plant, berries, and peanuts (Bryl et al., 2022). Japanese scientist Takaoka discovered and extracted RESV for the first time from a white hellebore in 1939 (Tsekovska et al., 2023). RESV became popular in 1991 on the “60 Minutes” CBS show when Drs. Michel de Lorgeril and Serge Renaud were featured about the study nicknamed the French Paradox or Red Wine Paradox. Based on the study, the French population exhibited a comparatively low prevalence of coronary heart disease due to their practice of consuming red wine, which hypothetically hinders the process of lipid peroxidation. Subsequently, RESV quickly gained widespread attention and sparked a significant amount of media coverage (Silva et al., 2022). The scientific studies discussed in the posts examined here reveal a wide range of bioactivities and significant potential health benefits of RESV. This finding appeared to contradict the prevailing notion that their diet, characterized by high fat intake, would contribute to adverse health outcomes (Ditano‐Vázquez et al., 2019).

RESV is predominantly present in the skin of red grapes and can be obtained through red wine as well. It is naturally synthesized by plants as a phytoalexin, which serves as a defensive mechanism against fungal infection, damage, stress, pathogens, and environmental stressors (Pannu & Bhatnagar, 2019). The levels of RESV in plants can vary depending on factors such as the plant species, climate, and growing conditions, although, RESV naturally exists as both cis and trans isomeric forms, the trans isomer is of particular interest due to its favorable health‐promoting properties, compared to the cis form, the trans form has a relatively higher bioavailability (Lubin et al., 2022).

Fresh grape skin is particularly rich in RESV, and during the past three decades, RESV has drawn a lot of attention for its favorable health effects, as it has attracted noteworthy interest in contemporary times owing to its plausible health advantages, such as its antioxidant, anti‐inflammatory, and anticancer attributes (Miguel et al., 2021). RESV is a promising compound for therapeutic interventions for cardiovascular diseases (CVDs), and neurodegenerative disorders. Its therapeutic ability to modulate several cellular pathways and affect various biological processes makes it a desirable bioactive compound against different chronic disorders (Thirumalaisamy et al., 2022). According to a number of studies, there is evidence to suggest that RESV may possess potential protective effects against a range of chronic diseases, including cancer (Honari et al., 2019), diabetes (Nowak et al., 2022), CVDs (Zhang et al., 2021), obesity (Thadhani, 2019), metabolic syndromes (Singh et al., 2019), kidney disorders (Alvarenga et al., 2022), and Alzheimer's disease (Tang et al., 2019). Moreover, previous preclinical investigations have demonstrated the potential of this intervention to augment cognitive abilities and attenuate the progression of aging (Terracina et al., 2022).

RESV being a potential antioxidant has been demonstrated to scavenge FRs and protect cells from oxidative damage. Various studies have investigated the possibility of encapsulating RESV in nanoparticles or using it as a nanocarrier. RESV has been proven as a promising choice for nutraceutical administration and functional food applications as studies have demonstrated that using it in nanoparticle formulations increases its bioavailability, absorption, and stability (Ahuja et al., 2020; Nguyen et al., 2022).

This review paper aims to provide an overview of the current state of knowledge regarding RESV, including its chemical nature, bioavailability, biological properties, health benefits based on clinical trials, and application of RESV in nanotechnology to enhance its bioavailability. Furthermore, this discussion will focus on the ongoing research about the potential therapeutic uses of RESV, while highlighting the specific areas that demand additional study.

## 2 CHEMICAL NATURE OF RESV

Natural polyphenolic antioxidant, RESV, is synthesized from 3 malonyl‐CoA molecules and 1 coumaric‐CoA molecule, as the method is catalyzed via stilbene synthase (Huang et al., 2020). Stilbene molecules are composed of an aromatic carbon skeleton of C_6_–C_2_–C_6_ (1,2‐diphenylethylene), of which RESV is a hydroxylated substance (Vestergaard & Ingmer, 2019). The trans‐RESV is the most abundant and has the greatest therapeutic potential because of its lower steric interruption (Shaito et al., 2020). During exposure to ultraviolet or solar radiation at wavelengths of 254 and 366 nm, trans may lose its stability and become cis‐isomeric (Fiod Riccio et al., 2020). RESV possesses a similar chemical structure to estrogen diethylstilbestrol, in which a styrene double bond links two phenol rings to form 3,4,5‐trihydroxystilbene (Yang et al., 2015). Ring A includes two hydroxyl (–OH) groups at C_3_ and C_5_, whereas ring B possesses one hydroxyl group at C_4_. The structure of RESV consists of m‐hydroxyquinone and 4′‐hydroxystyryl moieties including rings A and B (Chan et al., 2019).

In RESV, hydroxyl groups are unstable, since the double bond between C and C makes it sensitive to light, pH, and temperature (Tian & Liu, 2020). RESV shows stability at room temperature in acidic pH but rapidly degrades in the alkaline pH whereas, trans‐RESV in a liquid state could be stabilized by reducing pH, temperature, and limiting oxygen exposure (Zupančič et al., 2015). There are two monomers formed when RESV is decomposed, including resorcinol and phenol. When RESV is heated to its decomposition temperature (380°C), an additional reaction occurs which produces a new product. Analysis of the product structure reveals that it is mainly present among C‐C double bonds and the hydroxyl group (da Silva et al., 2017).

## 3 BIOAVAILABILITY OF RESV

The lipophilic properties of RESV contribute to its high absorption (Gambini et al., 2015). It has been identified that approximately 75% of RESV is orally absorbed when ingested by humans (Walle, 2011), and is primarily absorbed through transepithelial diffusion. The biosynthesis of trans‐RESV‐3‐O‐glucuronide and trans‐RESV‐3‐sulfate occurs extensively in the intestine and liver through glucuronic acid conjugation and sulfation, respectively, its bioavailability is less than 1% (Summerlin et al., 2015). The effectiveness of RESV may be diminished by its low bioavailability (Gambini et al., 2015). When given orally, RESV has poor bioavailability, which represents one of the main obstacles in translating its therapeutic properties. It is very difficult to sustain a therapeutic level of RESV in blood circulation as it is removed by the body in an immensely rapid manner (Kemper et al., 2022).

The bioavailability of RESV is limited as it metabolizes rapidly. A maximum plasma concentration of 1.48–2.48 ng/mL has been observed between 0.8 and 1.3 h following the first administration of 25, 50, 100, and 150 mg of RESV (Almeida et al., 2009). Oral administration of 400 mg RESV results in a plasma concentration of 47 mg/mL (Vaz‐da‐Silva et al., 2008), whereas 500 mg results in 71 mg/mL (Sergides et al., 2016).

The process of active transport facilitates the absorption of RESV metabolites, subsequently leading to their presence in the circulatory system, where they exhibit binding affinity toward lipoproteins and albumin. Upon interaction with cells possessing receptors for lipoproteins and albumin, these complexes facilitate the release of RESV into the cell. Subsequently, upon dissociation, RESV gains unhindered entry into the cell. Thus, these complexes serve as RESV reservoirs and aid in the distribution of the active compound (Pannu & Bhatnagar, 2019). Despite the rapid metabolism of RESV, the oral administration method is usually preferred, although plasma concentrations of RESV that do not undergo metabolism depend on the dosage (Chimento et al., 2019). The major metabolism of RESV occurs in the intestines and liver, where UDP‐glucuronosyltransferases and sulfotransferases are responsible for converting the compound into glucuronide and sulfate forms (Pannu & Bhatnagar, 2019; Sergides et al., 2016). After oral administration of RESV, the major RESV metabolites encountered are dihydro RESV, RESV‐3‐O‐sulfate, and RESV‐3‐O‐glucuronide conjugates that cause the low bioavailability of RESV (Peñalva et al., 2018).

## 4 HEALTH BENEFITS OF RESV

**FIGURE 1:**
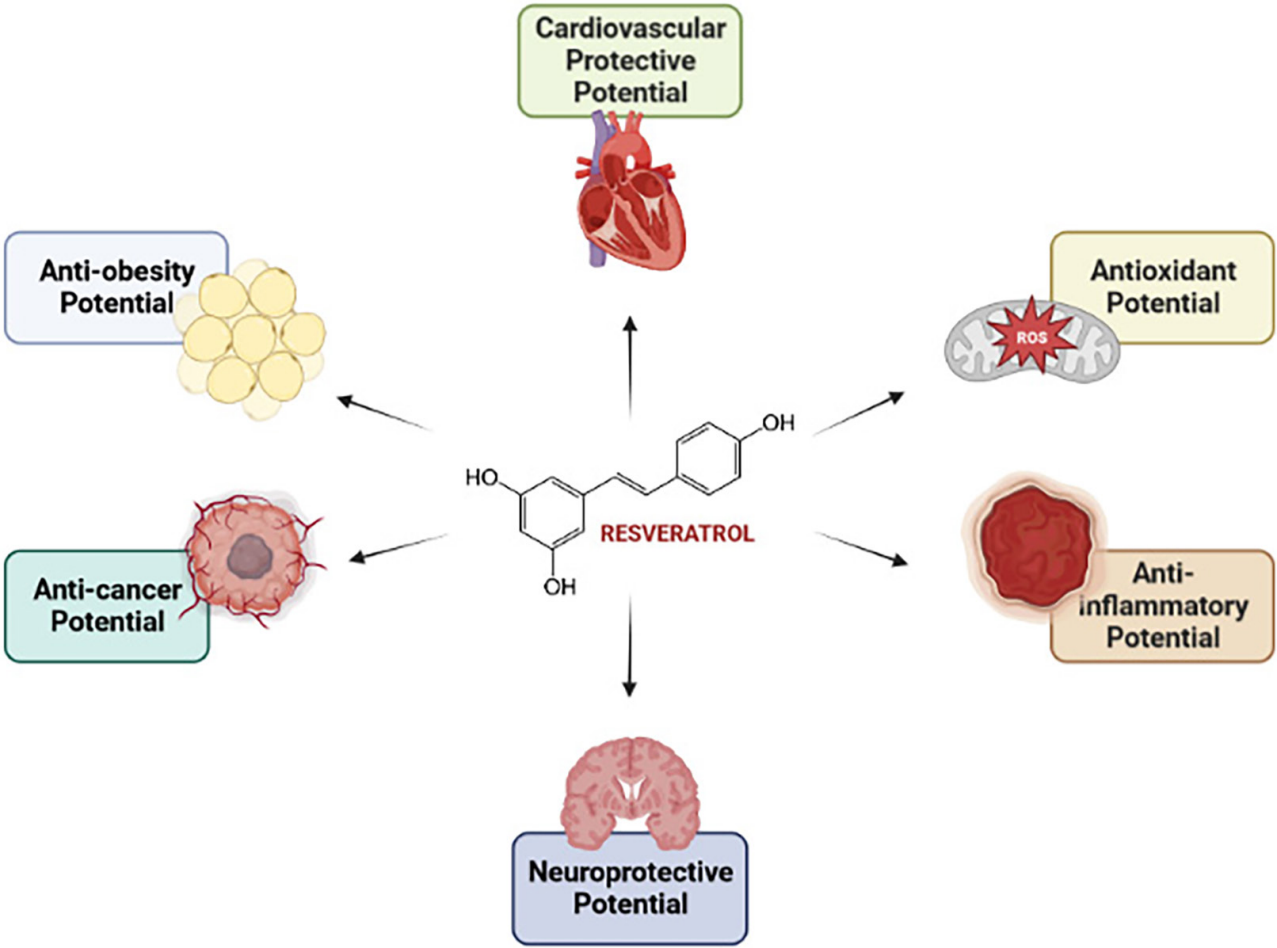
Reported health benefits associated with RESV consumption.

RESV has attracted great attention worldwide as it has been demonstrated to be one of the key components that contribute to human health. Potent antioxidants like RESV can help the body fight off dangerous FRs, which are known to speed up aging and cause several ailments (Lucarini et al., 2021; Pastor et al., 2019). As presented in Figure 1, RESV consumption can provide different health benefits.

RESV has been demonstrated to enhance heart health by lowering oxidative stress (OS) and inflammation in the body, consequently, the risk of cancer, inflammatory diseases, heart disease, and stroke may also be reduced (Zhang et al., 2021). In addition to preventing the synthesis of pro‐inflammatory cytokines including tumor necrosis factor and interleukin‐1 (IL‐1), RESV also reduced inflammation by stimulating the anti‐inflammatory enzymes (Ghanbari et al., 2021). Additionally, as an estrogen receptor (ER) ligand and an ER coregulator, sirtuin 1 (SIRT1) mediates the modulation of inflammatory responses by suppressing the transcription of interleukin‐6 (IL‐6). It has been demonstrated that RESV is able to upregulate SIRT1, decrease NF‐κB and the related cascades, and block PYD domains containing protein 3 (NALP3) activation. This allows it to manage both pro‐ and anti‐inflammatory cytokines and chemokines, as well as avoid inflammation (Banez et al., 2020; Battineni et al., 2021).

The immune system's reaction to pathogens like bacteria, viruses, and some harmful substances may also be modulated by RESV (Abedini et al., 2021). RESV has also been observed to decrease the survival of bacteria and decrease contagious pulmonary inflammation without observable host harm in vivo, resulting in attenuated diarrhea and inflammation in rotavirus‐infected piglets (Meng et al., 2023). Remarkably, it has been shown that RESV significantly increased immunological function in immunosuppressed animals, demonstrating a bidirectional regulatory influence on immunity (Song et al., 2022).

RESV has also been shown to have anticancer effects, which may aid in limiting the development and spread of cancer cells. Additionally, RESV has shown a significant reduction in fasting blood sugar, triglycerides (TGs), and heart rate, all of which are CVD risk factors (Battineni et al., 2021). Moreover, it has been demonstrated to enhance cognitive performance and shield the brain against deterioration brought on by aging. In addition to this, RESV has been observed to lessen brain inflammation and may influence antiaging properties by enhancing mitochondrial function and activating specific genes that increase durability (Yanez et al., 2019). RESV is useful in preventing several diseases, including kidney problems, Alzheimer's disease, and Parkinson's disease, according to several epidemiological investigations (Battineni et al., 2021; Donia & Khamis, 2021; Rana et al., 2022). Owing to the encouraging outcomes from experimental research, numerous clinical studies have also revealed RESV's effectiveness against a variety of disorders as presented in Table 1. A few of RESV's health‐promoting potentials are discussed below.

**TABLE 1:** Clinical studies on RESV supplementation to target different health issues.

Clinical condition	RESV dose	Study type	Treatment	Subjects	Results	References
To increase Bioavailability of RESV	Capsules (25, 50 and 100 mg)	Clinical trial	14 doses were given multiple times for 6 days after every 4 h	Healthy 20 men and 20 women	150 mg doses were observed as more effective, whereas bioavailability increases after the morning dose	Almeida et al. (2009)
	Caplets (0.5, 1, 2.5 and 5 g)	Repeat dose trial	1 caplet per day for 29 days	22 Healthy men and 18 Healthy women	Insulin‐like growth factor‐1 was decreased (*p* < .04) which may help in cancer chemo‐preventive activity	Brown et al. (2010)
	3 different types of wine	Clinical trial	4 weeks	12 healthy men from age 25 to 45 years	RESV was most efficiently absorbed (mean serum value 416–471 μg/L) as compared to other polyphenols in wine	Goldberg et al. (2003)
	Capsules (250/500 mg)	Double‐blind, placebo control trial	Once per day, for 3 separate days	Young Healthy 4 men and 20 women	After both doses, deoxyhemoglobin increases, also increases oxygen extraction, and modulates cerebral blood flow	Kennedy et al. (2010)
	Caplets (0.5–1 g)	Clinical trial	1 caplet per day	20 Colon and prostate cancer patients	Modulates gastrointestinal tract conditions to provoke anticarcinogenic effects (5% reduced tumor cell proliferation)	Patel et al. (2010)
	Oral and intravenous dose (25 mg)	Clinical trial	25 mg per day	6 Healthy Volunteers, 3 men and 3 women	Systemic bioavailability of RESV is very low (<5 ng/mL in plasma), excreted in urine	Walle et al. (2004)
	In 4 quartiles (0.14–0.054 mg)	Cross‐sectional study	Data was collected in more than 3 years	1162 men and 1456 women of 19–84 years	Reduced waist circumference, hypertriglyceridemia, and low HDL cholesterol	Sohrab et al. (2013)
CVD	Oral administration 500 mg/day	Randomized trial	30 days with caloric intake as 1000 kcal/day	Healthy and slightly overweight subjects, 24 men and 24 women	Low caloric intake and RESV increase Sirtuin 1 concentration in serum (*p* < .0001)	Mansur et al. (2017)
	Trans‐RESV capsules (150 mg/day)	Randomized, double‐blind, placebo‐control trial	Daily intake for 4 weeks	25 men and 20 women, slightly overweight and obese volunteer	Did not show significant results in improving endothelial function	van der Made et al. (2017)
	RESV capsule‐ GE‐RES, Stilvid® (350 mg/capsule)	Triple‐blind, randomized, placebo‐control trial	Daily intake of one capsule for 6 months	75 patients with primary CVD	No changes were observed in renal, hepatic, and thyroid function, RESV exerts cardioprotective effects	Tomé‐Carneiro et al. (2012)
	Oral consumption of RESV (20 mg/dose) + calcium fructoborate	Randomized, double‐blinded, control, clinical trial	20 mg/day for 60 days	166 patients with Angina pectoris	Showed beneficial effects (30.3%) with supplementation of calcium fructoborate against angina	Militaru et al. (2013)
	Oral consumption of RESV (400 mg/dose)	Clinical trial	400 mg/day for 1 month	44 healthy subjects	reduced fasting insulin concentration and Intercellular Adhesion Molecule expressions	Agarwal et al. (2013)
Cancer	Trans‐RESV (10 or 100 mg/day)	Clinical trial	50 mg two times a day for 12 weeks	39 women in postcancer	Decrease in methylation of the tumor suppressor genes (*p* = .047)	Zhu et al. (2012)
	RESV + grape powder (20 and 80 mg/ dose)	Clinical trial	20 and 80 mg/day for 14 days	8 patients with colon cancer	Showed significant effects on colon cancer patients, reduced CD133 and LGR5 in normal colonic mucosa (*p* < .03)	Nguyen et al. (2009)
Type 2 Diabetes	RESV Tablets (40 and 500 mg/day)	Double‐blind, randomized control trial	6 months	Type 2 diabetic patients, 126 men and 66 women	500 mg dose prevented bone density loss in diabetic patients (*p* = .001)	Bo et al. (2018)
	RESV capsules (1000 mg/capsule)	Randomized, double‐blind, placebo‐control trial	500 mg twice a day for 5 weeks	14 diabetic men	Did not show significant impacts on glycemic index and gastric emptying, energy intake, and body weight	Thazhath et al. (2016)
	RESV oral consumption (1 g/day)	Randomized, double‐blind, control trial	1 g per day for 45 days	66 diabetic patients	Reduced HbA1c serum levels (*p* < .04), urinary albumin excretion (*p* < .001), fasting blood glucose (*p* < .001), and insulin resistance	Sattarinezhad et al. (2019)
	RESV oral consumption (250 mg/day)	Clinical trial	250 mg per day for 3 months	62 diabetic patients	No effects were observed on HDL, LDL, and body weight, Improves glycemic control in the body	Bhatt et al. (2012)
Metabolic syndrome	RESV oral supplementation (150 and 1000 mg/day)	Randomized, double‐blind, placebo‐control trial	150 and 1000 mg/day for 16 weeks	24 patients	Did not show significant effects on glucose homeostasis, blood pressure, and inflammatory status, increased total cholesterol levels	Kjær et al. (2017)
	RESV oral supplementation (500 mg/dose)	Randomized, double‐blind, placebo‐control trial	500 mg thrice a day for 90 days	24 patients diagnosed with metabolic syndrome	Decreased total insulin secretion (*p* = .003), body weight (*p* = .007), waist circumference (*p* = .004) and fat mass (*p* = .001)	Méndez‐del Villar et al. (2014)
	RESV oral supplement (2 g/day)	Randomized, double‐blind, placebo‐control trial	1 g twice a day for 30 days	28 obese men	Altered glucose homeostasis, improves insulin resistance	Walker et al. (2019)
Alzheimer disease	RESV oral supplement (500 g/dose)	Randomized, double‐blind, placebo‐control trial	500 mg a day for 13 weeks ending with 1000 mg per day	119 patients	Overcome blood–brain barrier, causes nausea and diarrhea	Turner et al. (2015)
	RESV (5 mg/dose)	Randomized, double‐blind, placebo‐control trial	5 mg/day with 8 oz of grape juice ingested for 1 year	39 patients with mild to moderate Alzheimer's disease	Did not show significant impacts, however, a low dose of RESV is safe	Zhu et al. (2018)
Renal disorders	RESV (150 and 450 mg/dose)	Randomized, double‐blind, placebo‐control trial	150 and 450 mg/day for 12 weeks	72 peritoneal dialysis patients	Improves ultrafiltration in peritoneal dialysis (*p* = .003)	Lin et al. (2016)
	RESV (500 mg/dose)	Randomized, double‐blind, placebo‐control trial	500 mL per day for 4 weeks placebo	11 nondialyzed patients with chronic kidney disease	No changes were observed for pro‐inflammatory and antioxidant biomarkers	Saldanha et al. (2016)

### 4.1 Antioxidant potential

The potential health benefits of RESV have garnered significant attention in recent years, particularly its antioxidant properties. The potential of RESV to scavenge FRs and alleviate OS has been proven, highlighting its significance in the development and progression of several chronic diseases including cancer, CVDs, and neurological disorders. Moreover, research has shown that RESV can activate various cellular signaling pathways, which promote cellular health and longevity (Hu et al., 2019).

In a study conducted in 2018, Fu et al. demonstrated the antioxidative properties of RESV. Impact of RESV was studied on the suppression of inducible nitric oxide synthase (iNOS) expression and the mitogen‐activated protein kinase (p38‐MAPK) signaling pathway. Additionally, the study aimed to assess the potential protective effects of RESV administration against spinal cord reperfusion injury caused by heightened oxidative stress resulting from ischemia. After administering RESV therapy, the investigators observed a noteworthy reduction in plasma concentrations of nitrite and nitrate, as well as diminished mRNA and protein expressions of iNOS. Additionally, there was a significant drop in the phosphorylation of p38MAPK. Furthermore, the study provided evidence that the administration of RESV can effectively mitigate ischemic injury to the spinal cord. This protective effect is achieved through the inhibition of the iNOS/p38MAPK pathway, hence reducing the likelihood of organ dysfunction resulting from ischemia–reperfusion injury (Fu et al., 2018).

Deficiencies in wound healing arise from a multitude of intrinsic and extrinsic factors that contribute to the pathophysiology of the wound, culminating in significant morbidity and mortality rates globally (Oguntibeju, 2019). The process of wound healing is subject to perturbation by OS‐induced injuries, which have the potential to modify the entire healing course. Zhou et al. used human umbilical vein endothelial cells (HUVEC) for in vitro experiments to evaluate the effect of RESV on OS and cell proliferation. The findings of the study revealed that hydrogen peroxide‐induced OS undermines the ability of HUVEC to proliferate and migrate, whereas pretreatment with RESV mitigates this deleterious impact. In vivo experiments utilizing wound models demonstrated that RESV accelerated the wound healing process, conceivably via the attenuation of OS‐induced damage through the activation of nuclear factor erythroid 2‐related factor 2 (Nrf‐2) and manganese superoxide dismutase (Zhou et al., 2021).

A research investigation was conducted to assess the efficacy of two newly discovered analogs of RESV, specifically 4‐(E)‐(p‐tolylimino)‐methylbenzene‐1,2‐diol (TIMBD) and 4‐(E)‐(4‐hydroxyphenylimino)‐methylbenzene‐1,2‐diol (HPIMBD), in terms of their antioxidative properties in relation to breast cancer. Both of these chemicals are commonly referred to as HPIMBD and TIMBD. Based on the results obtained from the investigation, it was observed that both HPIMBD and TIMBD analogs exhibited no discernible indications of cytotoxicity toward nontumorigenic breast epithelial cells. Furthermore, it has been found that these analogs elicit activation of the antioxidant defense system through the nuclear factor erythroid 2 (Nrf2)‐dependent signaling pathway. This finding implies that these analogs may have potential use in regulating cellular antioxidant responses in the context of combating breast cancer (Chatterjee et al., 2018).

Santos et al. conducted a study to ascertain the antioxidant impact of RESV on human mononuclear cells (PBMC) under oxidative stress. The PBMCs were sourced from donors of different age groups. The chemiluminescence assay employed RESV at a dose of 5 micromolar, together with a stimulus containing 0.64% hydrogen peroxide, and inhibitors targeting the PKA, AkT/PKB, and MAPK signaling pathways. The senior group had a greater baseline generation of ROS compared to the middle‐aged group. RESV was effective in reducing ROS in both groups, but the middle‐aged group experienced a more pronounced decrease in ROS (Santos et al., 2021).

RESV can immediately eliminate peroxynitrite when it is exposed to authentic peroxynitrite in a system without cells. This strongly inhibits the nitration of bovine serum albumin by a factor of twenty, surpassing the effectiveness of N‐acetyl‐l‐cysteine. RESV inhibits the oxidation of LDL caused by peroxynitrite, as well as the cytotoxic effects created by peroxynitrite (Holthoff et al., 2010). RESV has been shown to greatly enhance the activation of eNOS via stimulating the membrane estrogen receptor. RESV facilitates the augmentation of endothelial nitric oxide (NO) production through various mechanisms, with this being just one of them. Moreover, outside the direct protective effects of endothelial NO, NO production mediated by estrogen receptors (ER) likely contributes to the increase in antioxidant proteins such as thioredoxin‐1 and heme oxygenase‐1 (HO1) that are stimulated by RESV (Xia et al., 2014). RESV increases mitochondrial biogenesis and reduces the amounts of ROS in mitochondria by inhibiting ROS formation and enhancing antioxidant defense systems, hence accelerating the detoxification of ROS. RESV enhances the synthesis of superoxide dismutase (SOD2) in a manner that relies on SIRT1. Elevating the level of SOD2 within mitochondria results in an augmentation of hydrogen peroxide (H_2_O_2_) generation. This H_2_O_2_ can effortlessly traverse mitochondrial membranes and disperse into the cytoplasm. Notably, treatment with RESV results in reduced cytoplasmic levels of H_2_O_2_. This could be due to enhanced H_2_O_2_ detoxification by GPx1 within the mitochondria and/or increased H_2_O_2_ inactivation by GPx1 and catalase in the cytoplasm. RESV possesses the capacity to enhance the activity of both antioxidant enzymes (Xia et al., 2017).

### 4.2 Anti‐inflammatory potential

The inflammatory reaction is a multistage process that involves various cell types and mediator signals (Lugrin et al., 2014). Damaged tissues or pathogenic infections trigger inflammation, which is the body's normal and crucial response to such signals (Tu et al., 2022).

**FIGURE 2:**
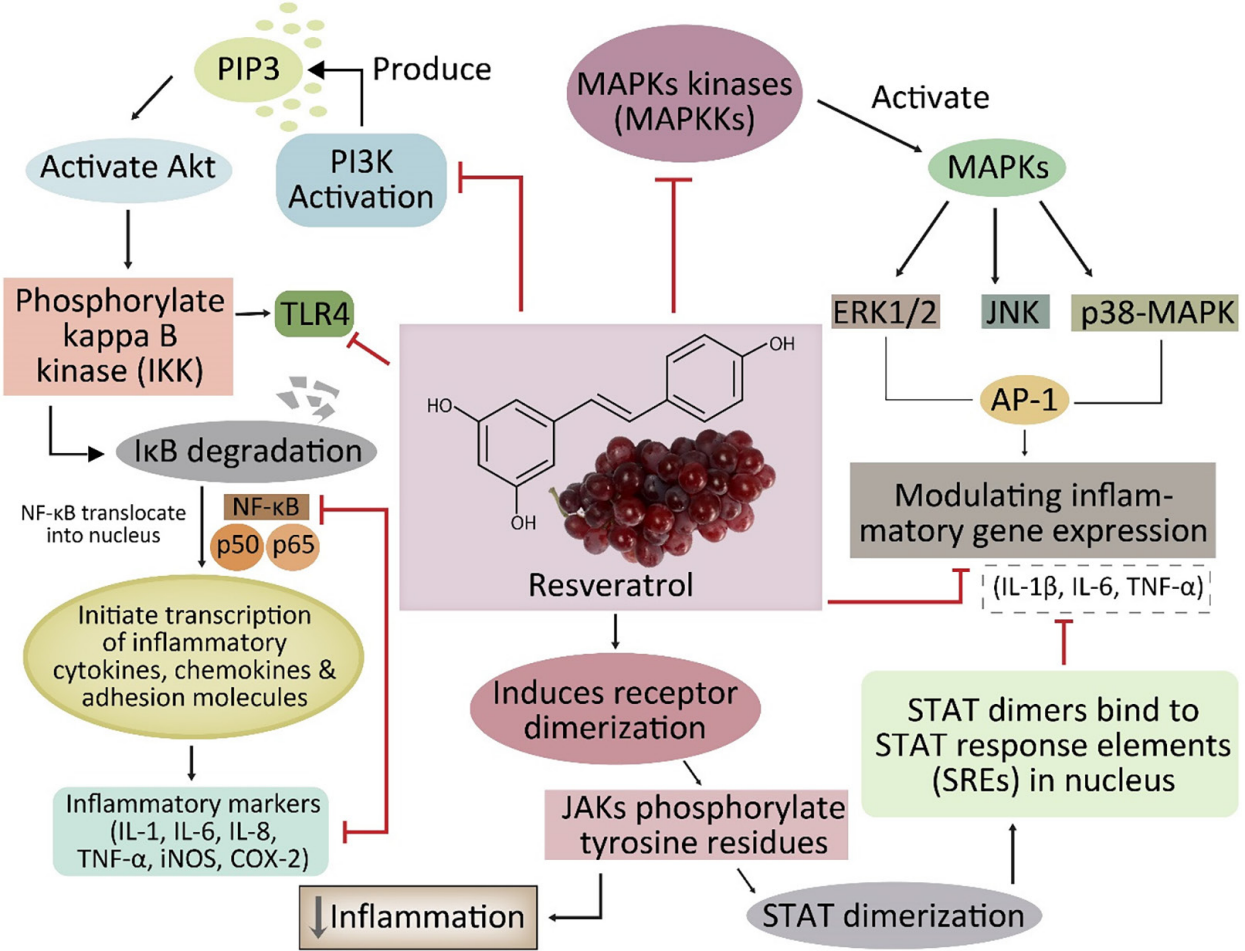
RESV and inflammatory parameters. Numerous cell signaling pathways are activated by inflammation. RESV directly inhibits the phosphoinositide 3‐kinases (PI3K)/Akt, mitogen‐activated protein kinases (MAPKs) signaling pathways which lead to NF‐κB activation that stimulate the production of several inflammatory markers. It suppresses the activation of NF‐κB directly through the facilitation of the degradation and phosphorylation of IκBα, one of the proteins associated with IκB. A downregulation of Janus kinase (JAK) is one of the effects of RESV on signal transducers and activators of transcription (STAT) protein phosphorylation and activation. The overall STAT signaling pathway could potentially be impacted by resveratrol by suppressing the activation of JAKs or other signaling molecules upstream of STAT proteins.

Downregulation of inflammatory responses is one of RESV's potential protective mechanisms as shown in Figure 2. Through downregulation of the toll‐like receptors (TLR‐4), NF‐κB, signal transducers and activators of transcription (STAT) signaling pathway, RESV exhibits anti‐inflammatory action both in vitro and in vivo compared to prompted microglial activation. By inhibiting these multiple mechanisms, RESV reduces the inflammation in rat models. Anti‐inflammatory properties of RESV include suppressing p38‐MAPK and NF‐κB pathways and lowering the serum levels of tumor necrosis factor‐alpha (TNF‐α), IL‐1, and IL‐6, which are primarily responsible for its positive benefits (Hsu et al., 2021). RESV was found to have a potent anti‐inflammatory effect in cell culture studies by reducing the synthesis of prostaglandin E2 (PGE2), TNF‐α, NO, IL‐1, and IL‐6, encouraging the release of IL‐10, repressing the TLR4 expression, and preventing the initiation of extracellular signal‐regulated protein kinases 1 & 2 (ERK1/2), Jun N‐terminal kinase (JNK), p38 and MAPK (Liu et al., 2020). Additionally, RESV blocks the activity of cyclooxygenases (COX), through which arachidonic acid converts to prostaglandins. As a result of this pathway's regulation, inflammation is reduced, raising the prospect that RESV may be used to treat inflammatory diseases (Berman et al., 2017).

Inflammatory response plays a significant role in the progression of acute kidney injury (AKI) (Inoue et al., 2017). Pro‐inflammatory cytokines (TNF‐α, IL‐1, IL‐6, and IL‐18) play an important role in the development and progression of ischemic AKI (Sharfuddin & Molitoris, 2011). Studies showed a substantial correlation between RESV and decreased levels of IL‐1, and IL‐6. Clinical medications depend heavily on the effects of dose and time response. No animal studies on the dose–response and time‐response effects of RESV in the treatment of AKI were reported (Cao et al., 2022). To reduce AKI induced by sepsis in a rat model, RESV upregulated SIRT1 expression, reduced the release of inflammatory molecules, and promoted NF‐κB‐P65 deacetylation (Gan et al., 2017).

The treatment with RESV inhibited the intercellular adhesion molecule 1 (ICAM‐1), IL‐1, iNOS, and mRNA expression in human coronary arterial endothelial cells stimulated by TNF‐α, was one of the results of numerous in vitro studies that demonstrated the anti‐inflammatory effects of RESV in cardiac tissue (Huang et al., 2017). RESV's anti‐inflammatory potential on CVDs attributed to several mechanisms, including reduction of monocyte adhesion to endothelial cells, suppression of NF‐кB and JAK (Janus kinase)/STAT signal pathways, upregulation of anti‐inflammatory cytokines, and inhibition of the production of pro‐inflammatory mediators like TNF‐α, IL‐1, and IL‐6 (Gal et al., 2021). Additionally, RESV administration for eight weeks was cardioprotective against myocardial infarction in rats inoculated with isoproterenol, a robust sympathetic agent. It is interesting to note that the protective effects of RESV were accompanied by a decrease in the pro‐inflammatory members of p38‐MAPK and ERK1/2, demonstrating that the control of these pro‐inflammatory pathways may be a factor in the therapeutic effects of RESV in cardiac disorders (Riba et al., 2017). In astrocytes exposed to lipopolysaccharides (LPS), RESV might in fact activate phosphatidylinositol 3‐kinase (PI3K)/Akt and Nrf2 pathways and inhibit NF‐κB and p38 MAPK (Bobermin et al., 2019).

Espinoza et al. investigated the influence of RESV on immunological cells. 1 g of RESV capsules were given daily to nine healthy men and women for twenty‐eight days. The outcomes of the research indicated that the administration of RESV directed to an elevation in the number of T cells present in the bloodstream, eventually resulting in a reduction in the levels of TNF‐α and monocyte chemoattractant protein 1, which are both pro‐inflammatory cytokines found in the plasma. Additionally, RESV increased plasma antioxidant capacity and subsequently reduced DNA damage‐causing OS markers (Espinoza et al., 2017). According to research, the treatment of RESV in a Crohn's disease (CD) rat model reduced inflammation and attenuated fibrosis. With the downregulation of insulin‐like growth factor 1 (IGF‐1) and procollagen mRNA expression, RESV significantly decreased the expression of inflammatory cytokines (IL‐1, IL‐6, and TNF‐α), profibrotic factors like transforming growth factor beta (TGF‐ β1) (Rahal et al., 2012).

Chronic obstructive pulmonary disease (COPD) is pathogenetically influenced by inflammation. RESV was administered by oral gavage to ten Wistar rats for 20 days (50 mg/kg) prior to smoking. Following a 30‐day investigative study, the researchers observed a reduction in inflammation and a reconstructed appearance of the small airways in the lungs. Treatment with RESV reduced IL‐6 and IL‐8 serum levels. The SIRT1/peroxisome proliferator‐activated receptor‐gamma coactivator (PGC‐1) signaling pathways may have been activated and upgraded as part of the mechanism (Wang et al., 2017). Moreover, these anti‐inflammatory effects of RESV can indirectly downregulate both respiratory and skeletal muscle damage in COPD patients (Beijers et al., 2018). RESV exerts its anti‐inflammatory properties through several pathways and intermediaries, thereby mitigating the pathophysiological effects of SARS‐CoV‐2 infection. It seems to have the capacity to inhibit the production of interleukins such as IL‐6, IL‐1β, and IL‐8, therefore decreasing inflammation (Akhondzadeh et al., 2020). RESV functions as a stimulator of the SIRT1 gene. SIRT1 is an enzyme associated with COVID‐19 due to its possession of the regulator NAD^+^. With advancing age, there is a decline in the levels of NAD^+^ in individuals. As the activation of SIRT1 relies on the presence of NAD^+^, reduced levels of NAD^+^ are likewise associated with lower SIRT1 activity. Consequently, the administration of nutritional supplements containing SIRT1 activators such as RESV has the potential to decrease the intensity of the disease (Miller et al., 2020).

### 4.3 Anti‐allergic potential

Allergy is a hyperactive response of the immune system to typically innocuous exogenous substances, primarily proteins, resulting in symptoms such as skin rash, sneezing, or mucous membrane inflammation. The global incidence of allergic disorders is substantial, with nearly 30% of the world's population experiencing one or more forms of allergic conditions. Clemens von Pirquet devised the word, “allergy” in the early 1900s. It refers to a group of diseases, such as allergic rhinitis, atopic dermatitis, asthma, food allergy, drug allergy, and anaphylaxis, a potentially fatal systemic response caused by mast cells (MCs) (Sikdar et al., 2023). MCs are a component of the innate immune system and play a pivotal role in acute allergic and inflammatory reactions by discharging inflammatory mediators such as histamine, cytokines, chemotactic factors, proteases, and arachidonic acid metabolites (AAMs), which act on a variety of inflammatory cells (Civelek et al., 2022).

Secondary plant substances, such as RESV, are natural compounds that have gained attention as potential nutraceuticals. Furthermore, in vitro and in vivo studies have demonstrated its anti‐allergic effects (Chen et al., 2020; Zhang et al., 2019). In a research study, the effect of RESV on mast cells was investigated in vivo using ovalbumin‐induced allergic enteritis and experimental colitis in IL‐10^−/−^ mice. RESV was administered to the mice via drinking water, and its effects were analyzed. The research discovered that RESV demonstrated inhibitory effects on the process of IgE‐dependent degranulation and the expression of pro‐inflammatory cytokines, including TNF‐α, in both IgE/2,4‐dinitrophenyl (DNP)‐activated and LPS‐activated mast cells generated from bone marrow. The results of this study indicate that RESV has anti‐allergic and anti‐inflammatory properties and could be beneficial in the prevention or treatment of ailments linked with mast cells (Bilotta et al., 2022).

Allergic rhinitis is a type I hypersensitivity reaction mediated by IgE in the nasal passages. ROS production is a key process in which thioredoxin‐interacting protein (TXNIP) plays a vital role. RESV is a known inhibitor of TXNIP, and therefore, Zhang et al. aimed to investigate the effect and mechanism of RESV on an ovalbumin‐induced mouse model of allergic rhinitis. The study revealed significant changes in sneezing, nasal rubbing, inflammatory cytokine levels, eosinophil counts, TXNIP expression, and the levels of OS markers such as malondialdehyde and superoxide dismutase in RESV‐treated mice compared to untreated allergic rhinitis mice. The findings suggest that RESV may effectively alleviate allergic rhinitis by inhibiting the TXNIP‐mediated OS pathway (Zhang et al., 2020).

Zhang et al. provided evidence that RESV exhibited a reduction in the discharge of β‐hexosaminidase and histamine within rat basophilic leukemia‐2H3 cells. These observations implied that RESV mitigated these reactions through a dual mechanism, involving the downregulation of serum ovalbumin‐specific IgE, histamine, mouse mast cell proteinase, and the depletion of dendritic cells, B cells, and mast cells in the spleen or mesenteric lymph node (Zhang et al., 2019).

The investigation conducted by Li et al. examined the possible protective properties of RESV against allergic rhinitis and elucidated the associated signaling pathways. The formation of an effective mouse model of allergic rhinitis was achieved by administering ovalbumin by intraperitoneal injection. The animals were administered different doses of RESV (5, 30, and 50 mg/kg) via their dietary intake. Based on the results, it was observed that therapy involving RESV exhibited a reduction in the frequency of sneezing and nasal rubbing in mice afflicted with allergic rhinitis. Consequently, there was a decrease in the synthesis of histamine, ovalbumin‐specific immunoglobulin E (IgE) and immunoglobulin G1 (IgG1), interleukin‐4 (IL‐4), and leukotriene C4 (LTC4). Additionally, there was a reduction in the number of inflammatory cells, including leucocytes, eosinophils, lymphocytes, and neutrophils, as well as a decrease in the secretion of various cytokines, such as tumor necrosis factor‐alpha (TNF‐α), interleukin‐6 (IL‐6), interleukin‐10 (IL‐10), interleukin‐5 (IL‐5), interleukin‐13 (IL‐13), and interleukin‐17 (IL‐17). The data presented in this study offer compelling evidence for the possible use of RESV as a therapeutic agent for the management of allergic rhinitis (Li et al., 2020).

### 4.4 Antidiabetic potential

Diabetes mellitus is classified as a persistent metabolic condition characterized by elevated levels of blood glucose. This condition arises from impaired insulin function and/or secretion, occurring during periods of fasting as well as after meals (Faisal et al., 2022). Extensive research has been conducted on the antidiabetic effects of RESV in both human and animal models with diabetes. It has been observed that by enhancing glucose metabolism and increasing insulin sensitivity, RESV may aid in lowering blood sugar levels (Hoca et al., 2023). The potential for managing diabetes mellitus using RESV, either in combination with antidiabetic medications or as a standalone treatment, is noteworthy. Tamimi et al. intended to investigate the potential synergistic effects of combining RESV and pioglitazone (PGZ) in the treatment of streptozotocin (STZ)‐induced diabetes. The findings of the study demonstrate that the combination of PGZ + RESV delivered the most favorable outcomes, as evidenced by statistically significant reductions in fasting blood glucose and HbA1c levels. Furthermore, this combination of factors also mitigated lipid abnormalities and the subsequent increase in atherogenic indicators (Tamimi et al., 2023).

The objective of another study project was to assess the potential of RESV to induce favorable modifications in the Goto‐Kakizaki (GK) rat, a naturally occurring model of diabetes that exhibits certain similarities to individuals with type 2 diabetes. GK rats were administered RESV orally at a dosage of 20 milligrams per kilogram of body weight every day for a duration of 10 weeks. Subsequently, an assessment of the patient's glucose tolerance was conducted. The implementation of RESV administration resulted in enhanced glucose tolerance, decreased fat formation in skeletal muscle, notable improvement in the morphology of pancreatic islets, and partial restoration of adiponectin and leptin levels in the bloodstream. Based on the results obtained from the study, the administration of RESV to GK rats exhibited a significant decrease in the prevalence of key indicators associated with diabetes (Szkudelska et al., 2019).

The study conducted by Movahed et al. aimed to assess the effectiveness of RESV in reducing hyperglycemia among individuals diagnosed with type 1 diabetes. A total of 13 patients diagnosed with Type 1 Diabetes, comprising individuals of both genders, took part in this clinical trial. Each patient was administered 500 mg capsules of RESV, twice daily, for a duration of 60 days. The findings of the study revealed that the administration of RESV for a duration of 60 days led to a noteworthy reduction in fasting blood glucose and hemoglobin A1c levels when compared to the initial measurements (Movahed et al., 2020).

### 4.5 Anti‐obesity potential

The global prevalence of obesity has reached epidemic proportions, with a steady increase observed annually. The observed phenomenon is strongly associated with a decrease in physical activity and the adoption of bad dietary practices driven by the process of modernization. Obesity significantly contributes to the development and advancement of metabolic syndrome within the human body (Faisal et al., 2022). Research conducted on animals has demonstrated that RESV can replicate the physiological consequences of calorie restriction by activating a protein called SIRT1. SIRT1 is a significant contributor to the control of cellular energy balance and the generation of new mitochondria. Previous research conducted on rodents has demonstrated the advantageous impacts of RESV administration on various physiological aspects, including mitochondrial function, glucose metabolism, body composition, and liver fat storage (de Ligt et al., 2015).

The study conducted by Wang et al. explored the potential of RESV as an anti‐obesity agent in mice that were fed a high‐fat diet. The aim of this study was to examine the potential correlation between the anti‐obesity properties of RESV and alterations in the gut microbiota and metabolic processes. The research findings indicated that the administration of RESV resulted in positive changes in the composition and function of the gut microbiota, as well as improvements in the oxidative status of the intestinal tract. These effects suggested that RESV may have prebiotic properties that can impact the overall health of the host. Furthermore, it was observed that RESV underwent biotransformation by gut bacteria, resulting in the production of 3‐hydroxyphenyl propionic acid and 4‐hydroxyphenyl acetic acid. These metabolites were found to possess the ability to mitigate fat storage (Wang et al., 2020).

Carpéné et al. conducted a study to examine the potential of RESV to restrict glucose utilization within the gut‐adipose tissue axis. The study revealed that RESV demonstrated a higher level of inhibitory efficacy against α‐glucosidase compared to pancreatic lipase activity. The administration of RESV resulted in a fast attenuation of glucose transport in fully developed adipocytes, achieved by antagonizing the actions of insulin and insulin‐like lipogenic substances. In a time frame of two hours, it demonstrated that RESV effectively hindered the process of glucose integration into lipids within adipocytes. Notably, this inhibitory effect remained unchanged even when the depletion of membrane cholesterol was taken into consideration (Carpéné et al., 2019). A comprehensive review incorporating meta‐analysis was conducted to examine the impact of RESV on weight loss. RESV leads to a considerable reduction in body weight, body mass index (BMI), waist circumference, and fat mass (Tabrizi et al., 2020).

### 4.6 Anticancer potential

Cancer poses the most severe threat to human life and well‐being, ranking as the second most deadly ailment after cardiovascular disease. Globally, cancer accounts for approximately 16% of all deaths. Regrettably, all currently available anticancer medications have exhibited limitations, including resistance and unwanted effects. Hence, it is imperative to devise novel anticancer medications that have been meticulously refined. RESV has generated significant interest as a potential and versatile anticancer medication due to its potential use in chemotherapy and chemoprevention for several types of cancers (Ahmadi & Ebrahimzadeh, 2020).

By comparing the effects of RESV on normal cell lines and cancer cell lines, Wu et al. found that RESV had a higher level of toxicity against cancer cells. RESV can induce apoptosis in cancer cells, and this impact is influenced by the duration and dosage of the treatment. Following an extensive analysis of transcriptome sequencing data, a total of 330 genes exhibiting substantial differences from each other were identified. Out of these genes, 103 genes were identified as being upregulated, while 227 genes were identified as being downregulated. Analysis of the transcriptome and quantitative real‐time PCR data revealed that the administration of RESV led to a substantial alteration in the expression of numerous genes linked to the cell cycle. The modifications in the stages of the cell cycle at different time intervals after treatment with RESV were revealed through further inquiry. It was determined that the cells were arrested in the S phase as evidenced by a rise in the proportion of cells in the S phase and a decrease in the proportion of cells in the G1/G0 phase. RESV can effectively hinder the growth of 4T1 cancer cells by impeding the cell cycle and inducing apoptosis (Wu et al., 2019).

To examine the impact and underlying mechanism of RESV on Triple Negative Breast Cancer (TNBC) cells, Liang et al. employed RNA sequencing (RNA‐seq). The data demonstrated a consistent decline in the survival rate of MDA‐MB‐231 cells with increasing concentration of RESV treatment. The RNA‐seq investigation demonstrated that the RESV medication largely affected genes involved in apoptosis and the p53 signaling pathway. Moreover, it was shown that POLD1 served as the principal agent of apoptosis in MDA‐MB‐231 cells subjected to the influence of RESV. The expression levels of full‐length PARP1, PCNA, and BCL‐2 were significantly decreased upon treatment of the cells with RESV. Conversely, there was a considerable increase in the levels of Cleaved‐PARP1 and Cleaved‐Caspase3 expression. The results of animal investigations have demonstrated that RESV has a significant inhibitory impact on the formation of live malignancies. The study's findings suggest that RESV has the potential to trigger apoptosis in TNBC cells by suppressing the expression of POLD1, hence activating the apoptotic pathway (Liang et al., 2021). After being exposed to 100 μM of RSV for 24–72 h, this specific process resulted in the release of cytochrome c and caspase activators derived from mitochondria. Additionally, it caused the activation of caspases and the stimulation of calpain, a protease that is activated by calcium. Ultimately, this process led to the demise of breast cancer cells via apoptosis (Madreiter‐Sokolowski et al., 2017).

Jin et al. conducted a study to assess the effects of RESV and doxorubicin (DOX) on epithelial–mesenchymal transitions (EMTs) and chemoresistance in MCF‐7/ADR breast cancer cells that had developed resistance to adriamycin (ADR). The researchers successfully showed that the combination of RESV and DOX had a significant impact on suppressing the proliferation and spread of MCF‐7/ADR cells. By promoting the expression of silent mating type information regulation 2 homolog 1 (SIRT1) and modulating the SIRT1/β‐catenin pathway, RESV can reverse the EMT process in MCF‐7/ADR cells. The upregulation of ubiquitin‐mediated proteolysis facilitated the degradation of β‐catenin by promoting the activity of increased SIRT1 (Jin et al., 2019).

Habibie et al. discovered that RESV triggers apoptosis in both murine and human melanoma cells, leading to detrimental effects on these cells. The stimulatory action of RESV on apoptosis of cells was attributed to the inhibition of survivin. RESV effectively decreased survivin expression at the transcriptional level by regulating the STAT3/β‐catenin pathway, employing a mechanistic method. In addition, administration of oral RESV at a dosage of 100 mg/kg/day in a mouse model of melanoma showed significant inhibition of tumor growth by effectively reducing the expression of surviving (Habibie et al., 2014). The prostate cancer cell lines PC3 and DU145 exhibited a decline in their ability to store Ca^2+^ in the endoplasmic reticulum (ER) and a decrease in store‐operated Ca^2+^ entry (SOCE) when treated with 100 μM RSV for 24 h. Consequently, ER stress was triggered, leading to autophagic cell death (Selvaraj et al., 2016).

Li et al. demonstrated that RESV can hinder the proliferation of HeLa cells and enhance apoptosis by acting through the intrinsic and p53 pathways. The researchers showed that when HeLa cells were treated with a concentration of 20 μmol/L of RESV, it caused the activation of caspase −3 and −9, an increase in BAX levels, and a decrease in the production of Bcl‐XL and Bcl‐2 proteins. Furthermore, cells that had been subjected to RESV exhibited elevated levels of the protein p53 (Li et al., 2018). Liu et al. conducted a separate investigation that showed RESV's capacity to hinder the survival of colorectal cancer cells and enhance the expression of p53 and its target genes, including BAX and PUMA. These genes play a crucial role in p53‐dependent apoptosis. Furthermore, it was discovered that cells subjected to RESV treatment had augmentation in the expression of SET domain‐containing lysine methyltransferase 7/9 (SET7/9), a protein that positively regulates p53 (Liu et al., 2019).

Another research study was conducted to ascertain whether MCF‐7 cells, which were treated with RESV before being exposed to heat or radiotherapy, were more prone to undergoing apoptosis. Various doses of RESV were administered to MCF‐7 cancer cells to achieve an IC50% result. Subsequently, the cells exposed to the specific concentration of RESV were then exposed to either radiation or heat. The viability of MCF‐7 cells may be reduced when they are subjected to either irradiation or heat. In terms of additional details, there was an increase in the regulation of the Bax and caspase genes, whereas the expression of the Bcl‐2 gene dropped. The addition of RESV greatly enhanced the impact of radiation and heat on MCF‐7 cells. According to the findings of this study, RESV can activate the regulation of genes that promote cell death and decrease the viability of MCF‐7 cells (Amini et al., 2021).

## 5 RESV AND GUT MICROBIOTA

RESV's limited absorption has led to the belief that its possible therapeutic benefits are attributed to its interaction with the gut. There is a notion suggesting that RESV may exert its health benefits by altering the composition of the gut microbiota as several mechanisms have been proposed to explain this phenomenon (Bird et al., 2017). The ingestion of RESV has been found to cause changes in the gut microbiota and enhance glucose homeostasis in obese and diabetic mice (Chaplin et al., 2018). Cai et al. investigated the potential of RESV to regulate the composition of the gut microbiome, intestinal barrier dysfunction, and the inflammatory response in the genetic db/db mice model. The aim was to identify if RESV has any beneficial effects in preventing diabetic nephropathy. RESV enhanced the functionality of the intestinal barrier, while also decreasing intestinal permeability and inflammation. The gut microbiota composition in db/db persons was found to be significantly distinct from that of the control group of db/m mice (Cai et al., 2020).

In another study, it was postulated that RESV possesses anti‐obesity advantages, mostly through the modulation of gut microbiota, leading to enhanced fat storage and metabolism. After administering RESV, the gut microbes, as well as glucose and lipid metabolism, are examined in live mice that were given a high‐fat diet (HF). When given to HF mice at a dosage of 200 milligrams per kilogram per day, RESV significantly reduces both body and visceral adipose weights, as well as lowers blood glucose and cholesterol levels. RESV can mitigate the imbalance of the gut microbiota induced by the high‐fat diet by increasing the proportion of *Bacteroidetes* compared to *Firmicutes*, which leads to a significant decrease in the proliferation of *Enterococcus faecalis* while promoting the growth of *Lactobacillus* and *Bifidobacterium*. Furthermore, RESV has been demonstrated to significantly increase the fasting‐induced adipose factor through its physiological actions (Qiao et al., 2014).

The study conducted by Most et al. examined the impact of RESV and epigallocatechin‐3‐gallate supplementation on the microbiota composition in the human gut. The researchers found that administering a supplement containing RESV and epigallocatechin‐3‐gallate concurrently for a duration of twelve weeks had the potential to significantly decrease the abundance of *Bacteroidetes* and exhibited a tendency to decrease the abundance of *Faecalibacterium prausnitzii* in overweight individuals. Nevertheless, the same patterns were not observed in females (Most et al., 2017). Sung and colleagues discovered that administering RESV to obese mice has the potential to reduce the prevalence of *Turicibacteraceae*, *Moryella, Lachnospiraceae*, and *Akkermansia*, while simultaneously increasing the prevalence of *Bacteroides* and *Parabacteroides* (Sung, Byrne, et al., 2017). Furthermore, the improved regulation of glucose levels, resulting from the transfer of fecal matter from healthy mice treated with RESV, gave further proof that the modification of the gut microbiota was the cause of the compound's antidiabetic effects (Sung, Kim, et al., 2017).

Several research studies have also reported the effect of gut microbiota on RESV, that is, its bioconversion. Etxeberria et al. employed liquid chromatography (LC) in conjunction with high‐resolution mass spectrometry (MS) to detect and analyze the microbial metabolites found in the fecal samples of Wistar rats that were given trans‐RESV as a supplement. Two primary metabolites, dihydro RESV and lunularin, were identified in these samples (Etxeberria et al., 2015). Bode and colleagues conducted controlled intervention research to investigate the bioconversion of trans‐RESV by the human gut flora. The subjects consisted of individuals in good health who received a single oral administration of 0.5 mg. In addition, the team performed in vitro fermentation studies. LC‐ultraviolet‐MS/MS detection revealed the presence of three metabolites: dihydroresveratrol, lunularin, and 3,4‐dihydroxy‐trans‐stilbene (Bode et al., 2013).

## 6 NANOPARTICLES‐BASED RESV DELIVERY

The science of nano‐delivery systems is rapidly developing and involves the use of nanoscale materials to provide diagnostic tools and therapeutic agents in a controlled manner to specific targeted sites (Patra et al., 2018). The field of nanotechnology exhibits considerable promise due to its capability to enhance the stability and solubility of naturally insoluble or less soluble materials. Additionally, the enhancement of oral bioavailability of the composites can be achieved by the promotion of their stability within biological systems (De Vries et al., 2018). Nanotechnology enhances the bioavailability of RESV by reducing the compound's size to the nanoscale. This reduction in size leads to an increased surface area of the compound, allowing for more efficient interaction with biological processes. Nanoparticles can protect RESV from degradation and metabolism, hence enhancing its targeted delivery. Furthermore, nanoparticles can be manipulated to control the discharge of RESV, leading to a sustained therapeutic impact. The utilization of nanotechnology in the administration of RESV enhances the bioavailability of the substance, mostly as a result of the synergistic impact of these components (Gowd et al., 2022).

**FIGURE 3:**
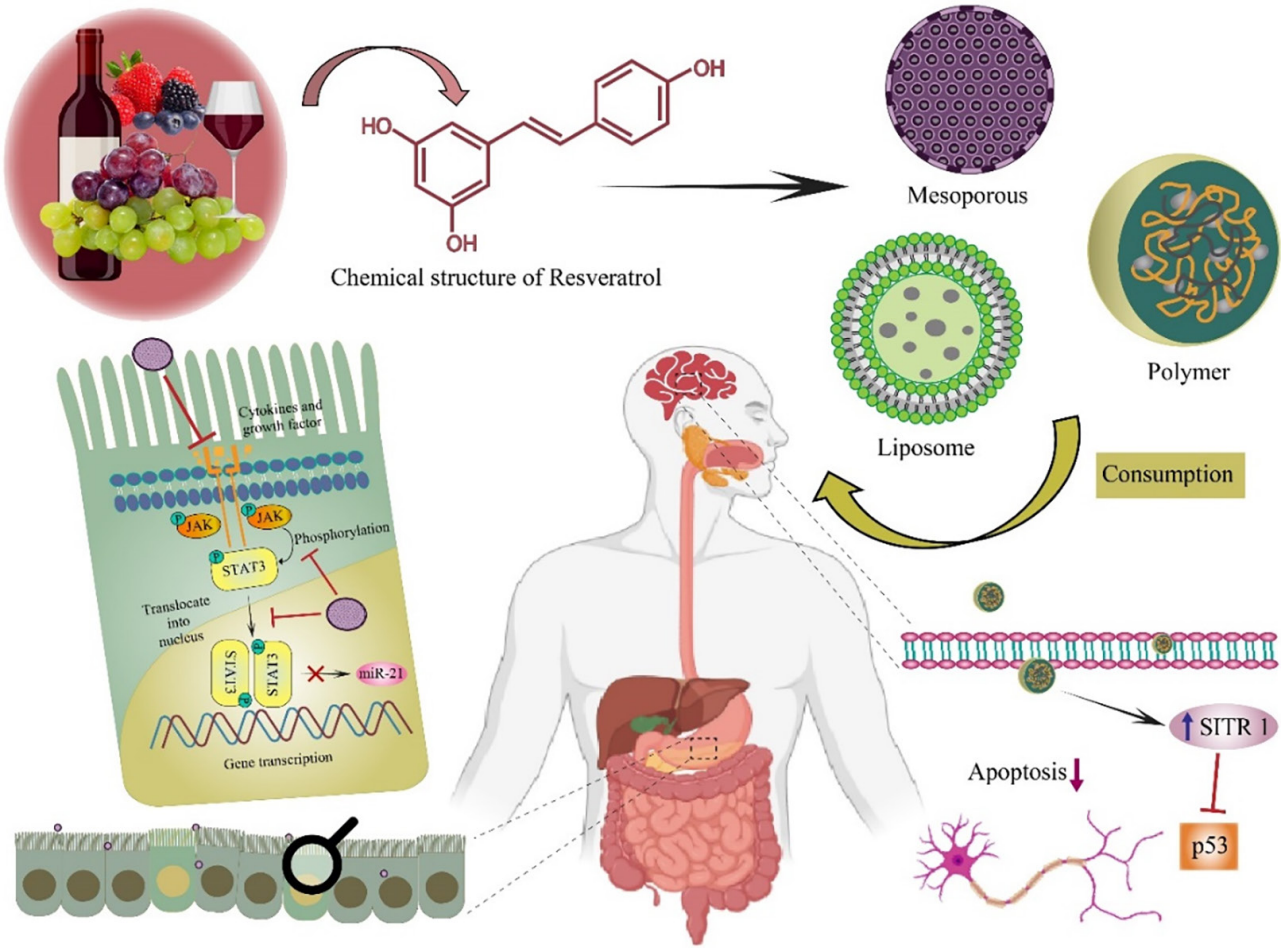
An overview of RESV sources, applications, and the types of nanoformulations used to deliver it, and their certain health benefits.

As an artificial vesicle with nanometer particle sizes and biocompatible nature, liposomes are a promising delivery system for drug delivery, allowing for increased drug solubility and bioavailability due to their size, amphiphilic nature, and biocompatibility (Yao et al., 2020). As shown in Figure 3, RESV has been delivered through different kinds of nanoparticles to obtain positive health outcomes. In addition to maintaining an intact membrane structure during digestion, RESV‐Liposomes were also capable of replicating intestinal conditions during hydrolysis. Furthermore, RESV‐LPS digests exhibited higher efficiency of cellular absorption and reduced ROS when compared with free RESV in the Caco‐2 cell uptake study (Xu et al., 2023). Different applications of RESV in nano‐encapsulation are presented in Table 2. A study found that RESV loaded in liposomes was released at slower rates than free RESV at pH 6.8 and pH 7.4, suggesting that its prolonged release profile could be enhanced by encapsulation. However, the pH 6.8 condition resulted in a significantly longer release than the pH 7.4 condition. According to this outcome, the liposomal formulation of RESV is useful in an acidic environment within the microenvironment compartment of the tumor (Dana et al., 2022).

**TABLE 2:** Application of RESV in Nano‐encapsulation.

Formulated nanomaterial	Encapsulating matrix	Nano‐encapsulation technique	Objectives	Outcomes	References
Trans‐RESV‐encapsulated nanoparticles (RNPs)	Chitosan	Coated in chitosan to prepare edible coatings	To develop a nutraceutical coating for the preservation of strawberry	The bioavailability of RESV increases in coated strawberry after oral administration, enhance preservation, reduces nutrient loss, and increase targeted delivery	Hu et al. (2023)
RESV microparticles	Starch	Electrohydrodynamic processing and emulsification	To encapsulate sensitive bioactive compounds as functional ingredients in functional foods	Antioxidant activity and bioaccessibility of RESV were increased after encapsulation in functional food	Silva (2023)
RESV‐loaded magnetic‐polymeric nanoparticles	Magnetic matrix (cobalt ferrite), Polymeric matrix (Chitosan + polyethylene glycol (PEG)	UV–VIS spectroscopy	To evaluate biological activities of magnetic‐polymeric nanoparticles	Reduced RESV degradation, increased encapsulation efficiency, decreased microbial growth	Buratto et al. (2023)
RESV Nano‐encapsulated supplements	—	—	To determine the anticancer effect of Nano‐encapsulated RESV against breast cancer	Decrease growth of tumor cells and oxidative stress	Sajadimajd et al. (2023)
RESV food‐grade nano‐emulsions	Soy lecithin + sugar esters + Tween 20 + glycerol monooleate	Nano‐emulsion	To determine the stability of RESV after encapsulation	RESV was efficiently absorbed by intestinal cell wall and not metabolized in GI tract	Sessa et al. (2011)
RESV‐casein micro‐ and nanoparticles	Casein extracted from Churpi‐Himalayan cheese	Emulsion‐freeze drying	To determine the viability and antioxidant activity of loaded RESV in casein	Anticancer and antioxidant properties of RESV were higher after encapsulation under simulated GI conditions, loaded nano‐ and microparticles can be employed in nutraceutical and food industry	Gani et al. (2022)
Polymeric liposomes	Pristine + cyclodextrin	Dual carrier approach	To access the targeted delivery of RESV	Co‐encapsulation increases targeted delivery of chemotherapeutic agent	Soo et al. (2016)
RESV Nanoparticles	Zein	Electro‐spraying	To improve bioavailability of RESV	Nano‐encapsulation of zein increases viability of RESV under simulated GI conditions	Jayan et al. (2019)
RESV‐PPI nanofibrils	Pea protein isolate (PPI)	Thermal method under acidic conditions	To improve stability of RESV and to increase anticancer and antioxidant properties of RESV	Enhance biological activities, improve chemical stability and water solubility of RESV	Yi et al. (2022)
RESV nanocapsules	Lipoic acid	Co‐nanoencapsulation via suspension method	To improve antioxidant properties for skin	Showed potential antioxidant effects, reduced cytotoxicity, increased stability	Davies et al. (2020)

Mesoporous silica nanoparticles (MSNs) possess a variety of beneficial morphological characteristics, including extensive precise surface area and pore volume, symmetrical shapes and sizes, colloidal stability, abundant surface chemistry, and a high dispersion (Kankala et al., 2020). Their distinctive structural characteristics make it easier to load the drug and then deliver it in a controlled manner to the target site (Mehmood et al., 2017). The RESV loading in MSN facilitated its amorphous transformation, which is a desired outcome aimed at enhancing the drug's solubility (Marinheiro et al., 2021). The antitumor impact of RESV‐loaded MSN indicated that they had a more effective anticancer effect than RESV alone treatment on gastric cancer therapy both in vivo and in vitro (Lin et al., 2021). RESV‐loaded MSN particles inhibited breast cancer progression more effectively than RESV alone by hindering the NF‐κB signaling pathway, indicating that MSN‐RESV can be used as adjuvant management for breast cancer with an improved efficacy. Phytochemicals can be used as a safe and new adjuvant treatment method to treat breast cancer, which is a very promising way of using phytochemicals in combination treatment (Gu & Fei, 2022).

By facilitating targeted delivery to specific tissues, polymeric nanoparticles have enormous potential to improve treatment efficacy. A biodegradable polymer could be used as a nanocarrier, as its properties can be intended to degrade in physiological conditions, and it can be engineered to exhibit prompted functionality when activated by an external source or at a particular site (Karlsson et al., 2018). Among these polymers, polyethylene glycol (PEG) and polylactic acid (PLA) were identified as harmless and practical elements for developing nano‐delivery systems and have received FDA approval for clinical usage (Mahapatro & Singh, 2011). A dose‐dependent suppression of CT26 colon cancer cell proliferation by RESV‐loaded polymeric nanoparticles was demonstrated in sulforhodamine B (SRB) assays, whereas minimal effects were found from empty polymeric nanoparticles. As a result of treatment with 20 and 40 mM of RESV‐encapsulated polymeric nanoparticles, the colony formation capacity of CT26 cells was significantly inhibited and apoptotic cell death was induced. The potential for inhibiting colony development and triggering apoptosis was even greater than that of free RESV (Jung et al., 2015). Studies on the pharmacokinetics and brain distribution in mice showed that polymeric nanoparticles considerably enhanced the bioavailability of paclitaxel and RESV polymeric nanoparticles compared to pure paclitaxel and RESV (Hussain et al., 2021).

Zhang et al. synthesized PEGylated nano‐liposomes for the simultaneous delivery of docetaxel (Doc) and RESV. This study aimed to assess the efficacy of combined drugs in the treatment of prostate cancer. Animal studies revealed that the co‐encapsulation of Doc/Res in liposomes significantly decreased tumor growth in PC3‐bearing Balb/c nude mice. This was evidenced by alterations in the parameters of cellular growth and apoptosis (Zhang et al., 2022). Rapamycin (RAP) and RESV coloaded liposomes (RAP‐RSV‐LIP) were developed as potential therapies for breast cancer. In vitro experiments revealed that RAP‐RSV‐LIP was infused in an estrogen receptor‐positive human breast cancer cell line (MCF‐7) at a rate of 34.2%. Furthermore, it exhibited enhanced cytotoxicity relative to free medications (Dos Reis et al., 2023).

## 7 CONCLUSION AND FUTURE DIRECTIONS

Resveratrol (RESV) demonstrates promising health benefits, particularly its antioxidant and anti‐inflammatory effects, as supported by clinical trials. Its ability to combat OS and inflammation suggests its potential in the prevention and management of several diseases. Nonetheless, RESV has shown significant attributes as a multimodal curative substance in promoting health and preventing chronic illnesses. RESV has been suggested to possess potential protective properties against various chronic ailments, including diabetes, CVDs, cancer, obesity, metabolic syndrome, kidney disorders, and Alzheimer's disease. The targeted delivery of RESV through nanoparticles has been utilized to address these ailments by targeting different inflammatory and oxidation pathways, immunomodulation, and neuro and cardiovascular protection. An additional comprehensive clinical study is necessary to validate the therapeutic advantages of RESV and ascertain its optimal dosage, potential long‐term consequences, and safety profile in various health conditions. It is highly recommended that both researchers and clinicians strengthen their attention to advancing targeted investigations, with a particular focus on novel nano‐delivery systems, to further improve the therapeutic effectiveness of resveratrol and make significant contributions to the field of health. Further investigation is necessary to elucidate the underlying processes accountable for the effects of RESV and to refine the administration protocols for certain contexts. Trial‐based advanced research is required in combinational nano‐delivery of RESV for the best possible health outcomes.

## AUTHOR CONTRIBUTIONS

**Zargham Faisal:** Conceptualization (equal); writing – original draft (equal); writing – review and editing (equal). **Aimen Mazhar:** Validation (equal); writing – original draft (equal). **Syeda Ayesha Batool:** Validation (equal); writing – original draft (equal). **Noor Akram:** Validation (equal); writing – original draft (equal). **Maleeha Hassan:** Data curation (equal); validation (equal). **Muhammad Usman Khan:** Data curation (equal); validation (equal). **Muhammad Afzaal:** Conceptualization (equal); supervision (equal); writing – review and editing (equal). **Usman Ul Hassan:** Formal analysis (equal); validation (equal). **Yasir Abbas Shah:** Validation (equal); writing – review and editing (equal). **Derese Tamiru Desta:** Supervision (equal); writing – review and editing (equal).

## CONFLICT OF INTEREST STATEMENT

The authors declare that they have no competing interests.

## Data Availability

Although enough data have been provided in the form of tables and figures, yet all authors declare if more data are required, the data will be provided on a request basis.

## References

[fsn33933-bib-0001] Abedini, E. , Khodadadi, E. , Zeinalzadeh, E. , Moaddab, S. R. , Asgharzadeh, M. , Mehramouz, B. , Dao, S. , & Samadi Kafil, H. (2021). A comprehensive study on the antimicrobial properties of resveratrol as an alternative therapy. Evidence‐based Complementary and Alternative Medicine, 2021, 8866311. 33815561 10.1155/2021/8866311 PMC7987421

[fsn33933-bib-0002] Agarwal, B. , Campen, M. J. , Channell, M. M. , Wherry, S. J. , Varamini, B. , Davis, J. G. , Baur, J. A. , & Smoliga, J. M. (2013). Resveratrol for primary prevention of atherosclerosis: Clinical trial evidence for improved gene expression in vascular endothelium. International Journal of Cardiology, 166(1), 246–248. 23098852 10.1016/j.ijcard.2012.09.027 PMC3646959

[fsn33933-bib-0003] Ahmadi, R. , & Ebrahimzadeh, M. A. (2020). Resveratrol—A comprehensive review of recent advances in anticancer drug design and development. European Journal of Medicinal Chemistry, 200, 112356. 32485531 10.1016/j.ejmech.2020.112356

[fsn33933-bib-0004] Ahuja, R. , Panwar, N. , Meena, J. , Singh, M. , Sarkar, D. P. , & Panda, A. K. (2020). Natural products and polymeric nanocarriers for cancer treatment: A review. Environmental Chemistry Letters, 18, 2021–2030.

[fsn33933-bib-0005] Akhondzadeh, F. , Astani, A. , Najjari, R. , Samadi, M. , Rezvani, M. E. , Zare, F. , Ranjbar, A. M. , & Safari, F. (2020). Resveratrol suppresses interleukin‐6 expression through activation of sirtuin 1 in hypertrophied H9c2 cardiomyoblasts. Journal of Cellular Physiology, 235(10), 6969–6977. 32026477 10.1002/jcp.29592

[fsn33933-bib-0006] Almeida, L. , Vaz‐da‐Silva, M. , Falcão, A. , Soares, E. , Costa, R. , Loureiro, A. I. , Fernandes‐Lopes, C. , Rocha, J. F. , Nunes, T. , Wright, L. , & Soares‐da‐Silva, P. (2009). Pharmacokinetic and safety profile of trans‐resveratrol in a rising multiple‐dose study in healthy volunteers. Molecular Nutrition & Food Research, 53(S1), S7–S15. 19194969 10.1002/mnfr.200800177

[fsn33933-bib-0007] Alvarenga, L. , Cardozo, L. F. , Leal, V. D. O. , Kemp, J. A. , Saldanha, J. F. , Ribeiro‐Alves, M. , Meireles, T. , Nakao, L. S. , & Mafra, D. (2022). Can resveratrol supplementation reduce uremic toxin plasma levels from the gut microbiota in nondialyzed patients with chronic kidney disease? Journal of Renal Nutrition, 32(6), 685–691. 35122992 10.1053/j.jrn.2022.01.010

[fsn33933-bib-0008] Amini, P. , Nodooshan, S. J. , Ashrafizadeh, M. , Eftekhari, S. M. , Aryafar, T. , Khalafi, L. , Musa, A. E. , Mahdavi, S. R. , Najafi, M. , & Farhood, B. (2021). Resveratrol induces apoptosis and attenuates proliferation of MCF‐7 cells in combination with radiation and hyperthermia. Current Molecular Medicine, 21(2), 142–150. 32436827 10.2174/1566524020666200521080953

[fsn33933-bib-0009] Banez, M. J. , Geluz, M. I. , Chandra, A. , Hamdan, T. , Biswas, O. S. , Bryan, N. S. , & Von Schwarz, E. R. (2020). A systemic review on the antioxidant and anti‐inflammatory effects of resveratrol, curcumin, and dietary nitric oxide supplementation on human cardiovascular health. Nutrition Research, 78, 11–26. 32428778 10.1016/j.nutres.2020.03.002

[fsn33933-bib-0010] Battineni, G. , Sagaro, G. G. , Chintalapudi, N. , Amenta, F. , Tomassoni, D. , & Tayebati, S. K. (2021). Impact of obesity‐induced inflammation on cardiovascular diseases (CVD). International Journal of Molecular Sciences, 22(9), 4798. 33946540 10.3390/ijms22094798 PMC8125716

[fsn33933-bib-0011] Beijers, R. J. , Gosker, H. R. , & Schols, A. M. (2018). Resveratrol for patients with chronic obstructive pulmonary disease: Hype or hope. Current Opinion in Clinical Nutrition and Metabolic Care, 21(2), 138–144. 29200030 10.1097/MCO.0000000000000444 PMC5811233

[fsn33933-bib-0012] Berman, A. Y. , Motechin, R. A. , Wiesenfeld, M. Y. , & Holz, M. K. (2017). The therapeutic potential of resveratrol: A review of clinical trials. npj Precision Oncology, 1(1), 35. 28989978 10.1038/s41698-017-0038-6 PMC5630227

[fsn33933-bib-0013] Bhatt, J. K. , Thomas, S. , & Nanjan, M. J. (2012). Resveratrol supplementation improves glycemic control in type 2 diabetes mellitus. Nutrition Research, 32(7), 537–541. 22901562 10.1016/j.nutres.2012.06.003

[fsn33933-bib-0014] Bilotta, S. , Arbogast, J. , Schart, N. , Frei, M. , & Lorentz, A. (2022). Resveratrol treatment prevents increase of mast cells in both murine OVA enteritis and IL‐10^−/−^ colitis. International Journal of Molecular Sciences, 23(3), 1213. 35163137 10.3390/ijms23031213 PMC8836010

[fsn33933-bib-0015] Bird, J. K. , Raederstorff, D. , Weber, P. , & Steinert, R. E. (2017). Cardiovascular and antiobesity effects of resveratrol mediated through the gut microbiota. Advances in Nutrition, 8(6), 839–849. 29141969 10.3945/an.117.016568 PMC5682996

[fsn33933-bib-0016] Bo, S. , Gambino, R. , Ponzo, V. , Cioffi, I. , Goitre, I. , Evangelista, A. , Ciccone, G. , Cassader, M. , & Procopio, M. (2018). Effects of resveratrol on bone health in type 2 diabetic patients. A double‐blind randomized‐controlled trial. Nutrition & Diabetes, 8(1), 51. 30237505 10.1038/s41387-018-0059-4 PMC6147949

[fsn33933-bib-0017] Bobermin, L. D. , Roppa, R. H. A. , & Quincozes‐Santos, A. (2019). Adenosine receptors as a new target for resveratrol‐mediated glioprotection. Biochimica et Biophysica Acta (BBA)—Molecular Basis of Disease, 1865(3), 634–647. 30611861 10.1016/j.bbadis.2019.01.004

[fsn33933-bib-0018] Bode, L. M. , Bunzel, D. , Huch, M. , Cho, G. S. , Ruhland, D. , Bunzel, M. , Bub, A. , Franz, C. M. , & Kulling, S. E. (2013). In vivo and in vitro metabolism of trans‐resveratrol by human gut microbiota. The American Journal of Clinical Nutrition, 97(2), 295–309. 23283496 10.3945/ajcn.112.049379

[fsn33933-bib-0019] Brown, V. A. , Patel, K. R. , Viskaduraki, M. , Crowell, J. A. , Perloff, M. , Booth, T. D. , Vasilinin, G. , Sen, A. , Schinas, A. M. , Piccirilli, G. , Brown, K. , Steward, W. P. , Gescher, A. J. , & Brenner, D. E. (2010). Repeat dose study of the cancer chemopreventive agent resveratrol in healthy volunteers: Safety, pharmacokinetics, and effect on the insulin‐like growth factor axis. Cancer Research, 70(22), 9003–9011. 20935227 10.1158/0008-5472.CAN-10-2364 PMC2982884

[fsn33933-bib-0020] Bryl, A. , Falkowski, M. , Zorena, K. , & Mrugacz, M. (2022). The role of resveratrol in eye diseases—A review of the literature. Nutrients, 14(14), 2974. 35889930 10.3390/nu14142974 PMC9317487

[fsn33933-bib-0021] Buratto, A. P. , Fontoura, B. H. , Carpes, S. T. , & Almeida, C. A. P. (2023). Preparation and characterization of chitosan/polyethylene glycol and cobalt ferrite magnetic‐polymeric nanoparticles as resveratrol carriers: Preparation, analytical methodology validation and in vitro applicability. Soft Materials, 21, 1–13.

[fsn33933-bib-0022] Cai, T. T. , Ye, X. L. , Li, R. R. , Chen, H. , Wang, Y. Y. , Yong, H. J. , Pan, M. L. , Lu, W. , Tang, Y. , Miao, H. , Snijders, A. M. , Mao, J. H. , Liu, X. Y. , Lu, Y. B. , & Ding, D. F. (2020). Resveratrol modulates the gut microbiota and inflammation to protect against diabetic nephropathy in mice. Frontiers in Pharmacology, 11, 1249. 32973502 10.3389/fphar.2020.01249 PMC7466761

[fsn33933-bib-0023] Cao, S. , Fu, X. , Yang, S. , & Tang, S. (2022). The anti‐inflammatory activity of resveratrol in acute kidney injury: A systematic review and meta‐analysis of animal studies. Pharmaceutical Biology, 60(1), 2088–2097. 36269038 10.1080/13880209.2022.2132264 PMC9590437

[fsn33933-bib-0024] Carpéné, C. , Les, F. , Cásedas, G. , Peiro, C. , Fontaine, J. , Chaplin, A. , Mercader, J. , & López, V. (2019). Resveratrol anti‐obesity effects: Rapid inhibition of adipocyte glucose utilization. Antioxidants, 8(3), 74. 30917543 10.3390/antiox8030074 PMC6466544

[fsn33933-bib-0025] Chan, E. W. C. , Wong, C. W. , Tan, Y. H. , Foo, J. P. Y. , Wong, S. K. , & Chan, H. T. (2019). Resveratrol and pterostilbene: A comparative overview of their chemistry, biosynthesis, plant sources and pharmacological properties. Journal of Applied Pharmaceutical Science, 9(7), 124–129.

[fsn33933-bib-0026] Chaplin, A. , Carpéné, C. , & Mercader, J. (2018). Resveratrol, metabolic syndrome, and gut microbiota. Nutrients, 10(11), 1651. 30400297 10.3390/nu10111651 PMC6266067

[fsn33933-bib-0027] Chatterjee, A. , Ronghe, A. , Padhye, S. B. , Spade, D. A. , Bhat, N. K. , & Bhat, H. K. (2018). Antioxidant activities of novel resveratrol analogs in breast cancer. Journal of Biochemical and Molecular Toxicology, 32(1), e21925. 10.1002/jbt.21925 28960787

[fsn33933-bib-0028] Chen, M. , Fu, Q. , Song, X. , Muhammad, A. , Jia, R. , Zou, Y. , Yin, L. , Li, L. , He, C. , Ye, G. , Lv, C. , Liang, X. , Huang, J. , Cui, M. , & Yin, Z. (2020). Preparation of resveratrol dry suspension and its immunomodulatory and anti‐inflammatory activity in mice. Pharmaceutical Biology, 58(1), 8–15. 31847682 10.1080/13880209.2019.1699123 PMC6968662

[fsn33933-bib-0029] Chimento, A. , De Amicis, F. , Sirianni, R. , Sinicropi, M. S. , Puoci, F. , Casaburi, I. , Saturnino, C. , & Pezzi, V. (2019). Progress to improve oral bioavailability and beneficial effects of resveratrol. International Journal of Molecular Sciences, 20(6), 1381. 30893846 10.3390/ijms20061381 PMC6471659

[fsn33933-bib-0030] Civelek, M. , Bilotta, S. , & Lorentz, A. (2022). Resveratrol attenuates mast cell mediated allergic reactions: Potential for use as a nutraceutical in allergic diseases? Molecular Nutrition & Food Research, 66(15), 2200170. 10.1002/mnfr.202200170 35598149

[fsn33933-bib-0031] da Silva, R. D. C. , Teixeira, J. A. , Nunes, W. D. G. , Zangaro, G. A. C. , Pivatto, M. , Caires, F. J. , & Ionashiro, M. (2017). Resveratrol: A thermoanalytical study. Food Chemistry, 237, 561–565. 28764035 10.1016/j.foodchem.2017.05.146

[fsn33933-bib-0032] Dana, P. , Thumrongsiri, N. , Tanyapanyachon, P. , Chonniyom, W. , Punnakitikashem, P. , & Saengkrit, N. (2022). Resveratrol loaded liposomes disrupt cancer associated fibroblast communications within the tumor microenvironment to inhibit colorectal cancer aggressiveness. Nanomaterials, 13(1), 107. 36616017 10.3390/nano13010107 PMC9824711

[fsn33933-bib-0033] Davies, S. , Contri, R. V. , Guterres, S. S. , Pohlmann, A. R. , & Guerreiro, I. C. K. (2020). Simultaneous nanoencapsulation of lipoic acid and resveratrol with improved antioxidant properties for the skin. Colloids and Surfaces B: Biointerfaces, 192, 111023. 32361374 10.1016/j.colsurfb.2020.111023

[fsn33933-bib-0034] de Ligt, M. , Timmers, S. , & Schrauwen, P. (2015). Resveratrol and obesity: Can resveratrol relieve metabolic disturbances? Biochimica et Biophysica Acta (BBA)—Molecular Basis of Disease, 1852(6), 1137–1144. 25446988 10.1016/j.bbadis.2014.11.012

[fsn33933-bib-0035] De Vries, K. , Strydom, M. , & Steenkamp, V. (2018). Bioavailability of resveratrol: Possibilities for enhancement. Journal of Herbal Medicine, 11, 71–77.

[fsn33933-bib-0036] Ditano‐Vázquez, P. , Torres‐Peña, J. D. , Galeano‐Valle, F. , Pérez‐Caballero, A. I. , Demelo‐Rodríguez, P. , Lopez‐Miranda, J. , Katsiki, N. , Delgado‐Lista, J. , & Alvarez‐Sala‐Walther, L. A. (2019). The fluid aspect of the Mediterranean diet in the prevention and management of cardiovascular disease and diabetes: The role of polyphenol content in moderate consumption of wine and olive oil. Nutrients, 11(11), 2833. 31752333 10.3390/nu11112833 PMC6893438

[fsn33933-bib-0037] Donia, T. , & Khamis, A. (2021). Management of oxidative stress and inflammation in cardiovascular diseases: Mechanisms and challenges. Environmental Science and Pollution Research, 28(26), 34121–34153. 33963999 10.1007/s11356-021-14109-9

[fsn33933-bib-0038] Dos Reis, L. R. , Luiz, M. T. , Sábio, R. M. , Marena, G. D. , Di Filippo, L. D. , Duarte, J. L. , Souza Fernandes, L. , Sousa Araújo, V. H. , Oliveira Silva, V. A. , & Chorilli, M. (2023). Design of rapamycin and resveratrol coloaded liposomal formulation for breast cancer therapy. Nanomedicine, 18(10), 789–801. 37199266 10.2217/nnm-2022-0227

[fsn33933-bib-0039] Espinoza, J. L. , Trung, L. Q. , Inaoka, P. T. , Yamada, K. , An, D. T. , Mizuno, S. , Nakao, S. , & Takami, A. (2017). The repeated administration of resveratrol has measurable effects on circulating T‐cell subsets in humans. Oxidative Medicine and Cellular Longevity, 2017, 6781872. 28546852 10.1155/2017/6781872 PMC5435979

[fsn33933-bib-0040] Etxeberria, U. , Arias, N. , Boqué, N. , Romo‐Hualde, A. , Macarulla, M. T. , Portillo, M. P. , Milagro, F. I. , & Martínez, J. A. (2015). Metabolic faecal fingerprinting of trans‐resveratrol and quercetin following a high‐fat sucrose dietary model using liquid chromatography coupled to high‐resolution mass spectrometry. Food & Function, 6(8), 2758–2767. 26156396 10.1039/c5fo00473j

[fsn33933-bib-0041] Faisal, Z. , Saeed, F. , Afzaal, M. , Akram, N. , Shah, Y. A. , Islam, F. , & Ateeq, H. (2022). Phytochemical profile and food applications of edible flowers: A comprehensive treatise. Journal of Food Processing and Preservation, 46(11), e17061.

[fsn33933-bib-0042] Fiod Riccio, B. V. , Fonseca‐Santos, B. , Colerato Ferrari, P. , & Chorilli, M. (2020). Characteristics, biological properties and analytical methods of trans‐resveratrol: A review. Critical Reviews in Analytical Chemistry, 50(4), 339–358. 31353930 10.1080/10408347.2019.1637242

[fsn33933-bib-0043] Fu, S. , Lv, R. , Wang, L. , Hou, H. , Liu, H. , & Shao, S. (2018). Resveratrol, an antioxidant, protects spinal cord injury in rats by suppressing MAPK pathway. Saudi Journal of Biological Sciences, 25(2), 259–266. 29472775 10.1016/j.sjbs.2016.10.019 PMC5815991

[fsn33933-bib-0044] Gal, R. , Deres, L. , Toth, K. , Halmosi, R. , & Habon, T. (2021). The effect of resveratrol on the cardiovascular system from molecular mechanisms to clinical results. International Journal of Molecular Sciences, 22(18), 10152. 34576315 10.3390/ijms221810152 PMC8466271

[fsn33933-bib-0045] Gambini, J. , Inglés, M. , Olaso, G. , Lopez‐Grueso, R. , Bonet‐Costa, V. , Gimeno‐Mallench, L. , Mas‐Bargues, C. , Abdelaziz, K. M. , Gomez‐Cabrera, M. C. , Vina, J. , & Borras, C. (2015). Properties of resveratrol: In vitro and in vivo studies about metabolism, bioavailability, and biological effects in animal models and humans. Oxidative Medicine and Cellular Longevity, 2015, 1–13. 10.1155/2015/837042 PMC4499410 26221416

[fsn33933-bib-0046] Gan, Y. , Tao, S. , Cao, D. , Xie, H. , & Zeng, Q. (2017). Protection of resveratrol on acute kidney injury in septic rats. Human & Experimental Toxicology, 36(10), 1015–1022. 27837177 10.1177/0960327116678298

[fsn33933-bib-0047] Gani, A. , Noor, N. , Gani, A. , Joseph‐Leenose‐Helen, J. , Shah, A. , & ul Ashraf, Z. (2022). Extraction of protein from churpi of yak milk origin: Size reduction, nutraceutical potential and as a wall material for resveratroleratrol. Food Bioscience, 46, 101612.

[fsn33933-bib-0048] Ghanbari, M. , Maragheh, S. M. , Aghazadeh, A. , Mehrjuyan, S. R. , Hussen, B. M. , Shadbad, M. A. , Dastmalchi, N. , & Safaralizadeh, R. (2021). Interleukin‐1 in obesity‐related low‐grade inflammation: From molecular mechanisms to therapeutic strategies. International Immunopharmacology, 96, 107765. 34015596 10.1016/j.intimp.2021.107765

[fsn33933-bib-0049] Goldberg, D. M. , Yan, J. , & Soleas, G. J. (2003). Absorption of three wine‐related polyphenols in three different matrices by healthy subjects. Clinical Biochemistry, 36(1), 79–87. 12554065 10.1016/s0009-9120(02)00397-1

[fsn33933-bib-0050] Gowd, V. , Jori, C. , Chaudhary, A. A. , Rudayni, H. A. , Rashid, S. , & Khan, R. (2022). Resveratrol and resveratrol nano‐delivery systems in the treatment of inflammatory bowel disease. The Journal of Nutritional Biochemistry, 109, 109101. 35777588 10.1016/j.jnutbio.2022.109101

[fsn33933-bib-0051] Gu, Y. , & Fei, Z. (2022). Mesoporous silica nanoparticles loaded with resveratrol are used for targeted breast cancer therapy. Journal of Oncology, 2022, 8471331. 36245986 10.1155/2022/8471331 PMC9553529

[fsn33933-bib-0052] Habibie, H. , Yokoyama, S. , Abdelhamed, S. , Awale, S. , Sakurai, H. , Hayakawa, Y. , & Saiki, I. (2014). Survivin suppression through STAT3/β‐catenin is essential for resveratrol‐induced melanoma apoptosis. International Journal of Oncology, 45(2), 895–901. 24946930 10.3892/ijo.2014.2480

[fsn33933-bib-0053] Hoca, M. , Becer, E. , & Vatansever, H. S. (2023). The role of resveratrol in diabetes and obesity associated with insulin resistance. Archives of Physiology and Biochemistry, 129(2), 555–561. 33719825 10.1080/13813455.2021.1893338

[fsn33933-bib-0054] Holthoff, J. H. , Woodling, K. A. , Doerge, D. R. , Burns, S. T. , Hinson, J. A. , & Mayeux, P. R. (2010). Resveratrol, a dietary polyphenolic phytoalexin, is a functional scavenger of peroxynitrite. Biochemical Pharmacology, 80(8), 1260–1265. 20599800 10.1016/j.bcp.2010.06.027 PMC2934873

[fsn33933-bib-0055] Honari, M. , Shafabakhsh, R. , Reiter, R. J. , Mirzaei, H. , & Asemi, Z. (2019). Resveratrol is a promising agent for colorectal cancer prevention and treatment: Focus on molecular mechanisms. Cancer Cell International, 19(1), 1–8. 31341423 10.1186/s12935-019-0906-y PMC6631492

[fsn33933-bib-0056] Hsu, Y. A. , Chen, C. S. , Wang, Y. C. , Lin, E. S. , Chang, C. Y. , Chen, J. J. Y. , Wu, M. Y. , Lin, H. J. , & Wan, L. (2021). Anti‐inflammatory effects of resveratrol on human retinal pigment cells and a myopia animal model. Current Issues in Molecular Biology, 43(2), 716–727. 34287272 10.3390/cimb43020052 PMC8929083

[fsn33933-bib-0057] Hu, Q. , Zhou, F. , Ly, N. K. , Ordyna, J. , Peterson, T. , Fan, Z. , & Wang, S. (2023). Development of multifunctional nanoencapsulated trans‐resveratrol/chitosan nutraceutical edible coating for strawberry preservation. ACS Nano, 17(9), 8586–8597. 37125693 10.1021/acsnano.3c01094

[fsn33933-bib-0058] Hu, Y. , Chen, D. , Zheng, P. , Yu, J. , He, J. , Mao, X. , & Yu, B. (2019). The bidirectional interactions between resveratrol and gut microbiota: An insight into oxidative stress and inflammatory bowel disease therapy. BioMed Research International, 2019, 5403761. 31179328 10.1155/2019/5403761 PMC6507241

[fsn33933-bib-0059] Huang, D. D. , Shi, G. , Jiang, Y. , Yao, C. , & Zhu, C. (2020). A review on the potential of resveratrol in prevention and therapy of diabetes and diabetic complications. Biomedicine & Pharmacotherapy, 125, 109767. 32058210 10.1016/j.biopha.2019.109767

[fsn33933-bib-0060] Huang, F. C. , Kuo, H. C. , Huang, Y. H. , Yu, H. R. , Li, S. C. , & Kuo, H. C. (2017). Anti‐inflammatory effect of resveratrol in human coronary arterial endothelial cells via induction of autophagy: Implication for the treatment of Kawasaki disease. BMC Pharmacology and Toxicology, 18, 1–8. 28069066 10.1186/s40360-016-0109-2 PMC5223384

[fsn33933-bib-0061] Hussain, T. , Paranthaman, S. , Rizvi, S. M. D. , Moin, A. , Gowda, D. V. , Subaiea, G. M. , Ansari, M. , & Alanazi, A. S. (2021). Fabrication and characterization of paclitaxel and resveratrol loaded soluplus polymeric nanoparticles for improved BBB penetration for glioma management. Polymers, 13(19), 3210. 34641026 10.3390/polym13193210 PMC8512154

[fsn33933-bib-0062] Inoue, T. , Tanaka, S. , & Okusa, M. D. (2017). Neuroimmune interactions in inflammation and acute kidney injury. Frontiers in Immunology, 8, 945. 28848551 10.3389/fimmu.2017.00945 PMC5552660

[fsn33933-bib-0063] Jayan, H. , Leena, M. M. , Sundari, S. S. , Moses, J. A. , & Anandharamakrishnan, C. (2019). Improvement of bioavailability for resveratrol through encapsulation in zein using electrospraying technique. Journal of Functional Foods, 57, 417–424.

[fsn33933-bib-0064] Jin, X. , Wei, Y. , Liu, Y. , Lu, X. , Ding, F. , Wang, J. , & Yang, S. (2019). Resveratrol promotes sensitization to doxorubicin by inhibiting epithelial‐mesenchymal transition and modulating SIRT1/β‐catenin signaling pathway in breast cancer. Cancer Medicine, 8(3), 1246–1257. 30697969 10.1002/cam4.1993 PMC6434195

[fsn33933-bib-0065] Jung, K. H. , Lee, J. H. , Park, J. W. , Quach, C. H. T. , Moon, S. H. , Cho, Y. S. , & Lee, K. H. (2015). Resveratrol‐loaded polymeric nanoparticles suppress glucose metabolism and tumor growth in vitro and in vivo. International Journal of Pharmaceutics, 478(1), 251–257. 25445992 10.1016/j.ijpharm.2014.11.049

[fsn33933-bib-0066] Kankala, R. K. , Han, Y. H. , Na, J. , Lee, C. H. , Sun, Z. , Wang, S. B. , Kimura, T. , Ok, Y. S. , Yamauchi, Y. , Chen, A. Z. , & Wu, K. C. W. (2020). Nanoarchitectured structure and surface biofunctionality of mesoporous silica nanoparticles. Advanced Materials, 32(23), 1907035. 10.1002/adma.201907035 32319133

[fsn33933-bib-0067] Karlsson, J. , Vaughan, H. J. , & Green, J. J. (2018). Biodegradable polymeric nanoparticles for therapeutic cancer treatments. Annual Review of Chemical and Biomolecular Engineering, 9, 105–127. 10.1146/annurev-chembioeng-060817-084055 PMC6215694 29579402

[fsn33933-bib-0068] Kemper, C. , Behnam, D. , Brothers, S. , Wahlestedt, C. , Volmar, C.‐H. , Bennett, D. , & Hayward, M. (2022). Safety and pharmacokinetics of a highly bioavailable resveratrol preparation (JOTROL TM). AAPS Open, 8(1), 11. 35789594 10.1186/s41120-022-00058-1 PMC9243782

[fsn33933-bib-0069] Kennedy, D. O. , Wightman, E. L. , Reay, J. L. , Lietz, G. , Okello, E. J. , Wilde, A. , & Haskell, C. F. (2010). Effects of resveratrol on cerebral blood flow variables and cognitive performance in humans: A double‐blind, placebo‐controlled, crossover investigation. The American Journal of Clinical Nutrition, 91(6), 1590–1597. 20357044 10.3945/ajcn.2009.28641

[fsn33933-bib-0070] Kjær, T. N. , Ornstrup, M. J. , Poulsen, M. M. , Stødkilde‐Jørgensen, H. , Jessen, N. , Jørgensen, J. O. L. , Richelsen, B. , & Pedersen, S. B. (2017). No beneficial effects of resveratrol on the metabolic syndrome: A randomized placebo‐controlled clinical trial. The Journal of Clinical Endocrinology & Metabolism, 102(5), 1642–1651. 28182820 10.1210/jc.2016-2160

[fsn33933-bib-0071] Li, J. , Wang, B. , Luo, Y. , Zhang, Q. , Bian, Y. , & Wang, R. (2020). Resveratrol‐mediated SIRT1 activation attenuates ovalbumin‐induced allergic rhinitis in mice. Molecular Immunology, 122, 156–162. 32361418 10.1016/j.molimm.2020.04.009

[fsn33933-bib-0072] Li, L. , Qiu, R. L. , Lin, Y. , Cai, Y. , Bian, Y. , Fan, Y. , & Gao, X. J. (2018). Resveratrol suppresses human cervical carcinoma cell proliferation and elevates apoptosis via the mitochondrial and p53 signaling pathways. Oncology Letters, 15(6), 9845–9851. 29928358 10.3892/ol.2018.8571 PMC6004645

[fsn33933-bib-0073] Liang, Z. J. , Wan, Y. , Zhu, D. D. , Wang, M. X. , Jiang, H. M. , Huang, D. L. , Luo, L. F. , Chen, M. J. , Yang, W. P. , Li, H. M. , & Wei, C. Y. (2021). Resveratrol mediates the apoptosis of triple negative breast cancer cells by reducing POLD1 expression. Frontiers in Oncology, 11, 569295. 33747905 10.3389/fonc.2021.569295 PMC7970754

[fsn33933-bib-0074] Lin, C. T. , Sun, X. Y. , & Lin, A. X. (2016). Supplementation with high‐dose trans‐resveratrol improves ultrafiltration in peritoneal dialysis patients: A prospective, randomized, double‐blind study. Renal Failure, 38(2), 214–221. 26727506 10.3109/0886022X.2015.1128236

[fsn33933-bib-0075] Lin, M. , Yao, W. , Xiao, Y. , Dong, Z. , Huang, W. , Zhang, F. , Zhou, X. , & Liang, M. (2021). Resveratrol‐modified mesoporous silica nanoparticle for tumor‐targeted therapy of gastric cancer. Bioengineered, 12(1), 6343–6353. 34506231 10.1080/21655979.2021.1971507 PMC8806839

[fsn33933-bib-0076] Liu, Y. , Liang, X. , Zou, Y. , Peng, Y. , McClements, D. J. , & Hu, K. (2020). Resveratrol‐loaded biopolymer core–shell nanoparticles: Bioavailability and anti‐inflammatory effects. Food & Function, 11(5), 4014–4025. 32322856 10.1039/d0fo00195c

[fsn33933-bib-0077] Liu, Z. , Wu, X. , Lv, J. , Sun, H. , & Zhou, F. (2019). Resveratrol induces p53 in colorectal cancer through SET7/9. Oncology Letters, 17(4), 3783–3789. 30881498 10.3892/ol.2019.10034 PMC6403518

[fsn33933-bib-0078] Lubin, B. C. R. , Inbar, N. , Pinkus, A. , Stanevsky, M. , Cohen, J. , Rahimi, O. , Anker, Y. , Shoseyov, O. , & Drori, E. (2022). Ecogeographic conditions dramatically affect trans‐resveratrol and other major phenolics' levels in wine at a semi‐arid area. Plants, 11(5), 629. 35270100 10.3390/plants11050629 PMC8912353

[fsn33933-bib-0079] Lucarini, M. , Durazzo, A. , Lombardi‐Boccia, G. , Souto, E. B. , Cecchini, F. , & Santini, A. (2021). Wine polyphenols and health: Quantitative research literature analysis. Applied Sciences, 11(11), 4762.

[fsn33933-bib-0080] Lugrin, J. , Rosenblatt‐Velin, N. , Parapanov, R. , & Liaudet, L. (2014). The role of oxidative stress during inflammatory processes. Biological Chemistry, 395(2), 203–230. 24127541 10.1515/hsz-2013-0241

[fsn33933-bib-0081] Madreiter‐Sokolowski, C. T. , Sokolowski, A. A. , & Graier, W. F. (2017). Dosis facit sanitatem—Concentration‐dependent effects of resveratrol on mitochondria. Nutrients, 9(10), 1117. 29027961 10.3390/nu9101117 PMC5691733

[fsn33933-bib-0082] Mahapatro, A. , & Singh, D. K. (2011). Biodegradable nanoparticles are excellent vehicle for site directed in‐vivo delivery of drugs and vaccines. Journal of Nanobiotechnology, 9, 1–11. 22123084 10.1186/1477-3155-9-55 PMC3238292

[fsn33933-bib-0083] Mansur, A. P. , Roggerio, A. , Goes, M. F. , Avakian, S. D. , Leal, D. P. , Maranhão, R. C. , & Strunz, C. M. (2017). Serum concentrations and gene expression of sirtuin 1 in healthy and slightly overweight subjects after caloric restriction or resveratrol supplementation: A randomized trial. International Journal of Cardiology, 227, 788–794. 28029409 10.1016/j.ijcard.2016.10.058

[fsn33933-bib-0084] Marinheiro, D. , Ferreira, B. J. , Oskoei, P. , Oliveira, H. , & Daniel‐da‐Silva, A. L. (2021). Encapsulation and enhanced release of resveratrol from mesoporous silica nanoparticles for melanoma therapy. Materials, 14(6), 1382. 33809119 10.3390/ma14061382 PMC8000002

[fsn33933-bib-0085] Mehmood, A. , Ghafar, H. , Yaqoob, S. , Gohar, U. F. , & Ahmad, B. (2017). Mesoporous silica nanoparticles: A review. Journal of Developing Drugs, 6(2).

[fsn33933-bib-0086] Méndez‐del Villar, M. , González‐Ortiz, M. , Martínez‐Abundis, E. , Pérez‐Rubio, K. G. , & Lizárraga‐Valdez, R. (2014). Effect of resveratrol administration on metabolic syndrome, insulin sensitivity, and insulin secretion. Metabolic Syndrome and Related Disorders, 12(10), 497–501. 25137036 10.1089/met.2014.0082

[fsn33933-bib-0087] Meng, Q. , Li, J. , Wang, C. , & Shan, A. (2023). Biological function of resveratrol and its application in animal production: A review. Journal of Animal Science and Biotechnology, 14(1), 1–23. 36765425 10.1186/s40104-022-00822-z PMC9921422

[fsn33933-bib-0088] Miguel, C. A. , Noya‐Riobó, M. V. , Mazzone, G. L. , Villar, M. J. , & Coronel, M. F. (2021). Antioxidant, anti‐inflammatory and neuroprotective actions of resveratrol after experimental nervous system insults. Special focus on the molecular mechanisms involved. Neurochemistry International, 150, 105188. 34536545 10.1016/j.neuint.2021.105188

[fsn33933-bib-0089] Militaru, C. , Donoiu, I. , Craciun, A. , Scorei, I. D. , Bulearca, A. M. , & Scorei, R. I. (2013). Oral resveratrol and calcium fructoborate supplementation in subjects with stable angina pectoris: Effects on lipid profiles, inflammation markers, and quality of life. Nutrition, 29(1), 178–183. 23153742 10.1016/j.nut.2012.07.006

[fsn33933-bib-0090] Miller, R. , Wentzel, A. R. , & Richards, G. A. (2020). COVID‐19: NAD^+^ deficiency may predispose the aged, obese and type2 diabetics to mortality through its effect on SIRT1 activity. Medical Hypotheses, 144, 110044. 32758884 10.1016/j.mehy.2020.110044 PMC7322475

[fsn33933-bib-0091] Most, J. , Penders, J. , Lucchesi, M. , Goossens, G. H. , & Blaak, E. E. (2017). Gut microbiota composition in relation to the metabolic response to 12‐week combined polyphenol supplementation in overweight men and women. European Journal of Clinical Nutrition, 71(9), 1040–1045. 28589947 10.1038/ejcn.2017.89

[fsn33933-bib-0092] Movahed, A. , Raj, P. , Nabipour, I. , Mahmoodi, M. , Ostovar, A. , Kalantarhormozi, M. , & Netticadan, T. (2020). Efficacy and safety of resveratrol in type 1 diabetes patients: A two‐month preliminary exploratory trial. Nutrients, 12(1), 161. 31935938 10.3390/nu12010161 PMC7019753

[fsn33933-bib-0093] Nguyen, A. V. , Martinez, M. , Stamos, M. J. , Moyer, M. P. , Planutis, K. , Hope, C. , & Holcombe, R. F. (2009). Results of a phase I pilot clinical trial examining the effect of plant‐derived resveratrol and grape powder on Wnt pathway target gene expression in colonic mucosa and colon cancer. Cancer Management and Research, 25–37. 21188121 PMC3004662

[fsn33933-bib-0094] Nguyen, D. D. , Luo, L. J. , Yang, C. J. , & Lai, J. Y. (2022). Highly retina‐permeating and long‐acting resveratrol/metformin nanotherapeutics for enhanced treatment of macular degeneration. ACS Nano, 17(1), 168–183. 36524981 10.1021/acsnano.2c05824

[fsn33933-bib-0095] Nowak, E. , Psiuk, D. , Rocka, A. , Dycha, N. , Jasielski, P. , Jasielska, F. , Madras, D. , & Rocka, E. (2022). Resveratrol impacts health in patients with diabetes mellitus and other metabolic conditions. Journal of Education, Health and Sport, 12(11), 341–346.

[fsn33933-bib-0096] Oguntibeju, O. O. (2019). Medicinal plants and their effects on diabetic wound healing. Veterinary World, 12(5), 653–663. 31327900 10.14202/vetworld.2019.653-663 PMC6584855

[fsn33933-bib-0097] Pannu, N. , & Bhatnagar, A. (2019). Resveratrol: From enhanced biosynthesis and bioavailability to multitargeting chronic diseases. Biomedicine & Pharmacotherapy, 109, 2237–2251. 30551481 10.1016/j.biopha.2018.11.075

[fsn33933-bib-0098] Pastor, R. F. , Restani, P. , Di Lorenzo, C. , Orgiu, F. , Teissedre, P. L. , Stockley, C. , Ruf, J. C. , Quini, C. I. , Garcìa Tejedor, N. , Gargantini, R. , Aruani, C. , Prieto, S. , Murgo, M. , Videla, R. , Penissi, A. , & Iermoli, R. H. (2019). Resveratrol, human health and winemaking perspectives. Critical Reviews in Food Science and Nutrition, 59(8), 1237–1255. 29206058 10.1080/10408398.2017.1400517

[fsn33933-bib-0099] Patel, K. R. , Brown, V. A. , Jones, D. J. , Britton, R. G. , Hemingway, D. , Miller, A. S. , West, K. P. , Booth, T. D. , Perloff, M. , Crowell, J. A. , Brenner, D. E. , Steward, W. P. , Gescher, A. J. , & Brown, K. (2010). Clinical pharmacology of resveratrol and its metabolites in colorectal cancer patients resveratrol in colorectal cancer patients. Cancer Research, 70(19), 7392–7399. 20841478 10.1158/0008-5472.CAN-10-2027 PMC2948608

[fsn33933-bib-0100] Patra, J. K. , Das, G. , Fraceto, L. F. , Campos, E. V. R. , Rodriguez‐Torres, M. D. P. , Acosta‐Torres, L. S. , Diaz‐Torres, L. A. , Grillo, R. , Swamy, M. K. , Sharma, S. , Habtemariam, S. , & Shin, H. S. (2018). Nano based drug delivery systems: Recent developments and future prospects. Journal of Nanobiotechnology, 16(1), 1–33. 30231877 10.1186/s12951-018-0392-8 PMC6145203

[fsn33933-bib-0101] Peñalva, R. , Morales, J. , González‐Navarro, C. J. , Larrañeta, E. , Quincoces, G. , Peñuelas, I. , & Irache, J. M. (2018). Increased oral bioavailability of resveratrol by its encapsulation in casein nanoparticles. International Journal of Molecular Sciences, 19(9), 2816. 30231546 10.3390/ijms19092816 PMC6163610

[fsn33933-bib-0102] Qiao, Y. , Sun, J. , Xia, S. , Tang, X. , Shi, Y. , & Le, G. (2014). Effects of resveratrol on gut microbiota and fat storage in a mouse model with high‐fat‐induced obesity. Food & Function, 5(6), 1241–1249. 24722352 10.1039/c3fo60630a

[fsn33933-bib-0103] Rahal, K. , Schmiedlin‐Ren, P. , Adler, J. , Dhanani, M. , Sultani, V. , Rittershaus, A. C. , Reingold, L. , Zhu, J. , McKenna, B. J. , Christman, G. M. , & Zimmermann, E. M. (2012). Resveratrol has antiinflammatory and antifibrotic effects in the peptidoglycan‐polysaccharide rat model of Crohn's disease. Inflammatory Bowel Diseases, 18(4), 613–623. 22431488 10.1002/ibd.21843 PMC3433226

[fsn33933-bib-0104] Rana, A. , Samtiya, M. , Dhewa, T. , Mishra, V. , & Aluko, R. E. (2022). Health benefits of polyphenols: A concise review. Journal of Food Biochemistry, 46(10), e14264. 35694805 10.1111/jfbc.14264

[fsn33933-bib-0105] Riba, A. , Deres, L. , Sumegi, B. , Toth, K. , Szabados, E. , & Halmosi, R. (2017). Cardioprotective effect of resveratrol in a postinfarction heart failure model. Oxidative Medicine and Cellular Longevity, 2017, 6819281. 29109832 10.1155/2017/6819281 PMC5646324

[fsn33933-bib-0106] Sajadimajd, S. , Aghaz, F. , Khazaei, M. , & Raygani, A. V. (2023). The anti‐cancer effect of resveratrol nano‐encapsulated supplements against breast cancer via the regulation of oxidative stress. Journal of Microencapsulation, 40, 318–329. 37017511 10.1080/02652048.2023.2198026

[fsn33933-bib-0107] Saldanha, J. F. , Leal, V. O. , Rizzetto, F. , Grimmer, G. H. , Ribeiro‐Alves, M. , Daleprane, J. B. , Carraro‐Eduardo, J. C. , & Mafra, D. (2016). Effects of resveratrol supplementation in Nrf2 and NF‐κB expressions in nondialyzed chronic kidney disease patients: A randomized, double‐blind, placebo‐controlled, crossover clinical trial. Journal of Renal Nutrition, 26(6), 401–406. 27523436 10.1053/j.jrn.2016.06.005

[fsn33933-bib-0108] Santos, M. A. , Franco, F. N. , Caldeira, C. A. , de Araújo, G. R. , Vieira, A. , Chaves, M. M. , & Lara, R. C. (2021). Antioxidant effect of resveratrol: Change in MAPK cell signaling pathway during the aging process. Archives of Gerontology and Geriatrics, 92, 104266. 33070070 10.1016/j.archger.2020.104266

[fsn33933-bib-0109] Sattarinezhad, A. , Roozbeh, J. , Yeganeh, B. S. , Omrani, G. R. , & Shams, M. (2019). Resveratrol reduces albuminuria in diabetic nephropathy: A randomized double‐blind placebo‐controlled clinical trial. Diabetes & Metabolism, 45(1), 53–59. 29983230 10.1016/j.diabet.2018.05.010

[fsn33933-bib-0110] Selvaraj, S. , Sun, Y. , Sukumaran, P. , & Singh, B. B. (2016). Resveratrol activates autophagic cell death in prostate cancer cells via downregulation of STIM1 and the mTOR pathway. Molecular Carcinogenesis, 55(5), 818–831. 25917875 10.1002/mc.22324 PMC4624064

[fsn33933-bib-0111] Sergides, C. , Chirilă, M. , Silvestro, L. , Pitta, D. , & Pittas, A. (2016). Bioavailability and safety study of resveratrol 500 mg tablets in healthy male and female volunteers. Experimental and Therapeutic Medicine, 11(1), 164–170. 26889234 10.3892/etm.2015.2895 PMC4726856

[fsn33933-bib-0112] Sessa, M. , Tsao, R. , Liu, R. , Ferrari, G. , & Donsì, F. (2011). Evaluation of the stability and antioxidant activity of nanoencapsulated resveratrol during in vitro digestion. Journal of Agricultural and Food Chemistry, 59(23), 12352–12360. 22026647 10.1021/jf2031346

[fsn33933-bib-0113] Shaito, A. , Posadino, A. M. , Younes, N. , Hasan, H. , Halabi, S. , Alhababi, D. , Al‐Mohannadi, A. , Abdel‐Rahman, W. M. , Eid, A. H. , Nasrallah, G. K. , & Pintus, G. (2020). Potential adverse effects of resveratrol: A literature review. International Journal of Molecular Sciences, 21(6), 2084. 32197410 10.3390/ijms21062084 PMC7139620

[fsn33933-bib-0114] Sharfuddin, A. A. , & Molitoris, B. A. (2011). Pathophysiology of ischemic acute kidney injury. Nature Reviews Nephrology, 7(4), 189–200. 21364518 10.1038/nrneph.2011.16

[fsn33933-bib-0115] Sikdar, A. , Suda, A. , Phatak, S. , Nivsarkar, S. , & Agarwal, R. (2023). Pattern of food allergen sensitivity amongst adult allergic rhinitis patients: A four year central Indian study. Indian Journal of Otolaryngology and Head & Neck Surgery, 75, 1–9. 10.1007/s12070-023-03544-4 PMC10188673 37206762

[fsn33933-bib-0116] Silva, P. , Portillo, M. P. , & Fernández‐Quintela, A. (2022). Resveratrol and wine: An overview of thirty years in the digital news. International Journal of Environmental Research and Public Health, 19(23), 15815. 36497888 10.3390/ijerph192315815 PMC9740773

[fsn33933-bib-0117] Silva, P. M. P. (2023). Micro‐nano encapsulation of melanoidins and resveratrol: Development of food functional ingredients in a circular economy approach.

[fsn33933-bib-0118] Singh, A. P. , Singh, R. , Verma, S. S. , Rai, V. , Kaschula, C. H. , Maiti, P. , & Gupta, S. C. (2019). Health benefits of resveratrol: Evidence from clinical studies. Medicinal Research Reviews, 39(5), 1851–1891. 30741437 10.1002/med.21565

[fsn33933-bib-0119] Sohrab, G. , Hosseinpour‐Niazi, S. , Hejazi, J. , Yuzbashian, E. , Mirmiran, P. , & Azizi, F. (2013). Dietary polyphenols and metabolic syndrome among Iranian adults. International Journal of Food Sciences and Nutrition, 64(6), 661–667. 23607642 10.3109/09637486.2013.787397

[fsn33933-bib-0120] Song, X. , Liu, L. , Peng, S. , Liu, T. , Chen, Y. , Jia, R. , Zou, Y. , Li, L. , Zhao, X. , Liang, X. , Tang, H. , & Yin, Z. (2022). Resveratrol regulates intestinal barrier function in cyclophosphamide‐induced immunosuppressed mice. Journal of the Science of Food and Agriculture, 102(3), 1205–1215. 34346509 10.1002/jsfa.11458

[fsn33933-bib-0121] Soo, E. , Thakur, S. , Qu, Z. , Jambhrunkar, S. , Parekh, H. S. , & Popat, A. (2016). Enhancing delivery and cytotoxicity of resveratrol through a dual nanoencapsulation approach. Journal of Colloid and Interface Science, 462, 368–374. 26479200 10.1016/j.jcis.2015.10.022

[fsn33933-bib-0122] Summerlin, N. , Soo, E. , Thakur, S. , Qu, Z. , Jambhrunkar, S. , & Popat, A. (2015). Resveratrol nanoformulations: Challenges and opportunities. International Journal of Pharmaceutics, 479(2), 282–290. 25572692 10.1016/j.ijpharm.2015.01.003

[fsn33933-bib-0123] Sung, M. M. , Byrne, N. J. , Robertson, I. M. , Kim, T. T. , Samokhvalov, V. , Levasseur, J. , Soltys, C. L. , Fung, D. , Tyreman, N. , Denou, E. , Jones, K. E. , Seubert, J. M. , Schertzer, J. D. , & Dyck, J. R. (2017). Resveratrol improves exercise performance and skeletal muscle oxidative capacity in heart failure. American Journal of Physiology. Heart and Circulatory Physiology, 312(4), H842–H853. 28159807 10.1152/ajpheart.00455.2016

[fsn33933-bib-0124] Sung, M. M. , Kim, T. T. , Denou, E. , Soltys, C. L. M. , Hamza, S. M. , Byrne, N. J. , Masson, G. , Park, H. , Wishart, D. S. , Madsen, K. L. , Schertzer, J. D. , & Dyck, J. R. (2017). Improved glucose homeostasis in obese mice treated with resveratrol is associated with alterations in the gut microbiome. Diabetes, 66(2), 418–425. 27903747 10.2337/db16-0680

[fsn33933-bib-0125] Szkudelska, K. , Deniziak, M. , Hertig, I. , Wojciechowicz, T. , Tyczewska, M. , Jaroszewska, M. , & Szkudelski, T. (2019). Effects of resveratrol in Goto‐Kakizaki rat, a model of type 2 diabetes. Nutrients, 11(10), 2488. 31623226 10.3390/nu11102488 PMC6836277

[fsn33933-bib-0126] Tabrizi, R. , Tamtaji, O. R. , Lankarani, K. B. , Akbari, M. , Dadgostar, E. , Dabbaghmanesh, M. H. , Kolahdooz, F. , Shamshirian, A. , Momen‐Heravi, M. , & Asemi, Z. (2020). The effects of resveratrol intake on weight loss: A systematic review and meta‐analysis of randomized controlled trials. Critical Reviews in Food Science and Nutrition, 60(3), 375–390. 30421960 10.1080/10408398.2018.1529654

[fsn33933-bib-0127] Tamimi, L. N. , Zakaraya, Z. , Hailat, M. , Abu Dayyih, W. , Daoud, E. , Abed, A. , Saadh, M. J. , Majeed, B. , Abumansour, H. , Aburumman, A. , Majeed, J. M. , & Hamad, M. (2023). Anti‐diabetic effect of cotreatment with resveratrol and pioglitazone in diabetic rats. European Review for Medical and Pharmacological Sciences, 27, 325–332. 36647881 10.26355/eurrev_202301_30879

[fsn33933-bib-0128] Tang, Y. W. , Shi, C. J. , Yang, H. L. , Cai, P. , Liu, Q. H. , Yang, X. L. , Kong, L. Y. , & Wang, X. B. (2019). Synthesis and evaluation of isoprenylation‐resveratrol dimer derivatives against Alzheimer's disease. European Journal of Medicinal Chemistry, 163, 307–319. 30529634 10.1016/j.ejmech.2018.11.040

[fsn33933-bib-0129] Terracina, S. , Petrella, C. , Francati, S. , Lucarelli, M. , Barbato, C. , Minni, A. , Ralli, M. , Greco, A. , Tarani, L. , Fiore, M. , & Ferraguti, G. (2022). Antioxidant intervention to improve cognition in the aging brain: The example of hydroxytyrosol and resveratrol. International Journal of Molecular Sciences, 23(24), 15674. 36555317 10.3390/ijms232415674 PMC9778814

[fsn33933-bib-0130] Thadhani, V. M. (2019). Resveratrol in management of diabetes and obesity: Clinical applications, bioavailability, and nanotherapy. In F. A. Badria (Ed.), Resveratrol—Adding life to years, not adding years to life (pp. 139–156).

[fsn33933-bib-0131] Thazhath, S. S. , Wu, T. , Bound, M. J. , Checklin, H. L. , Standfield, S. , Jones, K. L. , Horowitz, M. , & Rayner, C. K. (2016). Administration of resveratrol for 5 wk has no effect on glucagon‐like peptide 1 secretion, gastric emptying, or glycemic control in type 2 diabetes: A randomized controlled trial. The American Journal of Clinical Nutrition, 103(1), 66–70. 26607942 10.3945/ajcn.115.117440

[fsn33933-bib-0132] Thirumalaisamy, R. , Bhuvaneswari, M. , Haritha, S. , Jeevarathna, S. , Janani, K. S. , & Suresh, K. (2022). Curcumin, naringenin and resveratrol from natural plant products hold promising solution for modern world diseases—A recent review. South African Journal of Botany, 151, 567–580.

[fsn33933-bib-0133] Tian, B. , & Liu, J. (2020). Resveratrol: A review of plant sources, synthesis, stability, modification and food application. Journal of the Science of Food and Agriculture, 100(4), 1392–1404. 31756276 10.1002/jsfa.10152

[fsn33933-bib-0134] Tomé‐Carneiro, J. , Gonzálvez, M. , Larrosa, M. , García‐Almagro, F. J. , Avilés‐Plaza, F. , Parra, S. , Yáñez‐Gascón, M. J. , Ruiz‐Ros, J. A. , García‐Conesa, M. T. , Tomás‐Barberán, F. A. , & Espín, J. C. (2012). Consumption of a grape extract supplement containing resveratrol decreases oxidized LDL and a po B in patients undergoing primary prevention of cardiovascular disease: A triple‐blind, 6‐month follow‐up, placebo‐controlled, randomized trial. Molecular Nutrition & Food Research, 56(5), 810–821. 22648627 10.1002/mnfr.201100673

[fsn33933-bib-0135] Tsekovska, R. , Kirov, K. , Bozhinov, A. S. , Gatev, E. , Ivanov, I. , Niwa, T. , Mironova, R. , & Handzhyiski, Y. (2023). Antiglycation properties of resveratrol and glucosamine. Journal of Chemical Technology and Metallurgy, 58(2), 270–274.

[fsn33933-bib-0136] Tu, Z. , Zhong, Y. , Hu, H. , Shao, D. , Haag, R. , Schirner, M. , Lee, J. , Sullenger, B. , & Leong, K. W. (2022). Design of therapeutic biomaterials to control inflammation. Nature Reviews Materials, 7(7), 557–574. 35251702 10.1038/s41578-022-00426-z PMC8884103

[fsn33933-bib-0137] Turner, R. S. , Thomas, R. G. , Craft, S. , van Dyck, C. , Mintzer, J. , Reynolds, B. A. , Brewer, J. B. , Rissman, R. A. , Raman, R. , Aisen, P. S. , & Alzheimer's Disease Cooperative Study . (2015). A randomized, double‐blind, placebo‐controlled trial of resveratrol for Alzheimer disease. Neurology, 85(16), 1383–1391. 26362286 10.1212/WNL.0000000000002035 PMC4626244

[fsn33933-bib-0138] van der Made, S. M. , Plat, J. , & Mensink, R. P. (2017). Trans‐resveratrol supplementation and endothelial function during the fasting and postprandial phase: A randomized placebo‐controlled trial in overweight and slightly obese participants. Nutrients, 9(6), 596. 28604618 10.3390/nu9060596 PMC5490575

[fsn33933-bib-0139] Vaz‐da‐Silva, M. , Loureiro, A. I. , Falcao, A. , Nunes, T. , Rocha, J. F. , Fernandes‐Lopes, C. , Soares, E. , Wright, L. , Almeida, L. , & Soares‐da‐Silva, P. (2008). Effect of food on the pharmacokinetic profile of trans‐resveratrol. International Journal of Clinical Pharmacology and Therapeutics, 46(11), 564–570. 19000554 10.5414/cpp46564

[fsn33933-bib-0140] Vestergaard, M. , & Ingmer, H. (2019). Antibacterial and antifungal properties of resveratrol. International Journal of Antimicrobial Agents, 53(6), 716–723. 30825504 10.1016/j.ijantimicag.2019.02.015

[fsn33933-bib-0141] Walker, J. M. , Eckardt, P. , Aleman, J. O. , da Rosa, J. C. , Liang, Y. , Iizumi, T. , Etheve, S. , Blaser, M. J. , Breslow, L. J. , & Holt, P. R. (2019). The effects of trans‐resveratrol on insulin resistance, inflammation, and microbiota in men with the metabolic syndrome: A pilot randomized, placebo‐controlled clinical trial. Journal of Clinical and Translational Research, 4(2), 122. 30873501 PMC6412609

[fsn33933-bib-0142] Walle, T. (2011). Bioavailability of resveratrol. Annals of the New York Academy of Sciences, 1215(1), 9–15. 21261636 10.1111/j.1749-6632.2010.05842.x

[fsn33933-bib-0143] Walle, T. , Hsieh, F. , DeLegge, M. H. , Oatis, J. E. , & Walle, U. K. (2004). High absorption but very low bioavailability of oral resveratrol in humans. Drug Metabolism and Disposition, 32(12), 1377–1382. 15333514 10.1124/dmd.104.000885

[fsn33933-bib-0144] Wang, P. , Gao, J. , Ke, W. , Wang, J. , Li, D. , Liu, R. , Jia, Y. , Wang, X. , Chen, X. , Chen, F. , & Hu, X. (2020). Resveratrol reduces obesity in high‐fat diet‐fed mice via modulating the composition and metabolic function of the gut microbiota. Free Radical Biology and Medicine, 156, 83–98. 32305646 10.1016/j.freeradbiomed.2020.04.013

[fsn33933-bib-0145] Wang, X. L. , Li, T. , Li, J. H. , Miao, S. Y. , & Xiao, X. Z. (2017). The effects of resveratrol on inflammation and oxidative stress in a rat model of chronic obstructive pulmonary disease. Molecules, 22(9), 1529. 28895883 10.3390/molecules22091529 PMC6151812

[fsn33933-bib-0146] Wu, H. , Chen, L. , Zhu, F. , Han, X. , Sun, L. , & Chen, K. (2019). The cytotoxicity effect of resveratrol: Cell cycle arrest and induced apoptosis of breast cancer 4T1 cells. Toxins, 11(12), 731. 31847250 10.3390/toxins11120731 PMC6950385

[fsn33933-bib-0147] Xia, N. , Daiber, A. , Förstermann, U. , & Li, H. (2017). Antioxidant effects of resveratrol in the cardiovascular system. British Journal of Pharmacology, 174(12), 1633–1646. 27058985 10.1111/bph.13492 PMC5446570

[fsn33933-bib-0148] Xia, N. , Förstermann, U. , & Li, H. (2014). Resveratrol and endothelial nitric oxide. Molecules, 19(10), 16102–16121. 25302702 10.3390/molecules191016102 PMC6270738

[fsn33933-bib-0149] Xu, X. , Tian, M. , Deng, L. , Jiang, H. , Han, J. , Zhen, C. , Huang, L. , & Liu, W. (2023). Structural degradation and uptake of resveratrol‐encapsulated liposomes using an in vitro digestion combined with Caco‐2 cell absorption model. Food Chemistry, 403, 133943. 36191420 10.1016/j.foodchem.2022.133943

[fsn33933-bib-0150] Yanez, M. , Jhanji, M. , Murphy, K. , Gower, R. M. , Sajish, M. , & Jabbarzadeh, E. (2019). Nicotinamide augments the anti‐inflammatory properties of resveratrol through PARP1 activation. Scientific Reports, 9(1), 10219. 31308445 10.1038/s41598-019-46678-8 PMC6629694

[fsn33933-bib-0151] Yang, T. , Wang, L. , Zhu, M. , Zhang, L. , & Yan, L. (2015). Properties and molecular mechanisms of resveratrol: A review. Die Pharmazie—An International Journal of Pharmaceutical Sciences, 70(8), 501–506. 26380517

[fsn33933-bib-0152] Yao, H. , Lu, H. , Zou, R. , Chen, X. , & Xu, H. (2020). Preparation of encapsulated resveratrol liposome thermosensitive gel and evaluation of its capability to repair sciatic nerve injury in rats. Journal of Nanomaterials, 2020, 1–13.

[fsn33933-bib-0154] Yi, J. , He, Q. , Peng, G. , & Fan, Y. (2022). Improved water solubility, chemical stability, antioxidant and anticancer activity of resveratrol via nanoencapsulation with pea protein nanofibrils. Food Chemistry, 377, 131942. 34990943 10.1016/j.foodchem.2021.131942

[fsn33933-bib-0155] Zhang, L. , Lin, Z. , Chen, Y. , Gao, D. , Wang, P. , Lin, Y. , Wang, Y. , Wang, F. , Han, Y. , & Yuan, H. (2022). Co‐delivery of docetaxel and resveratrol by liposomes synergistically boosts antitumor efficiency against prostate cancer. European Journal of Pharmaceutical Sciences, 174, 106199. 35533965 10.1016/j.ejps.2022.106199

[fsn33933-bib-0156] Zhang, L. X. , Li, C. X. , Kakar, M. U. , Khan, M. S. , Wu, P. F. , Amir, R. M. , Dai, D. F. , Naveed, M. , Li, Q. Y. , Saeed, M. , Shen, J. Q. , Rajput, S. A. , & Li, J. H. (2021). Resveratrol (RV): A pharmacological review and call for further research. Biomedicine & Pharmacotherapy, 143, 112164. 34649335 10.1016/j.biopha.2021.112164

[fsn33933-bib-0157] Zhang, W. , Tang, R. , Ba, G. , Li, M. , & Lin, H. (2020). Anti‐allergic and anti‐inflammatory effects of resveratrol via inhibiting TXNIP‐oxidative stress pathway in a mouse model of allergic rhinitis. World Allergy Organization Journal, 13(10), 100473. 33133334 10.1016/j.waojou.2020.100473 PMC7586246

[fsn33933-bib-0158] Zhang, Y. F. , Liu, Q. M. , Gao, Y. Y. , Liu, B. , Liu, H. , Cao, M. J. , Yang, X. W. , & Liu, G. M. (2019). Attenuation of allergic responses following treatment with resveratrol in anaphylactic models and IgE‐mediated mast cells. Food & Function, 10(4), 2030–2039. 30907398 10.1039/c9fo00077a

[fsn33933-bib-0159] Zhou, X. , Ruan, Q. , Ye, Z. , Chu, Z. , Xi, M. , Li, M. , Hu, W. , Guo, X. , Yao, P. , & Xie, W. (2021). Resveratrol accelerates wound healing by attenuating oxidative stress‐induced impairment of cell proliferation and migration. Burns, 47(1), 133–139. 33288327 10.1016/j.burns.2020.10.016

[fsn33933-bib-0160] Zhu, C. W. , Grossman, H. , Neugroschl, J. , Parker, S. , Burden, A. , Luo, X. , & Sano, M. (2018). A randomized, double‐blind, placebo‐controlled trial of resveratrol with glucose and malate (RGM) to slow the progression of Alzheimer's disease: A pilot study. Alzheimer's & Dementia: Translational Research & Clinical Interventions, 4, 609–616. 10.1016/j.trci.2018.09.009 PMC6240843 30480082

[fsn33933-bib-0161] Zhu, W. , Qin, W. , Zhang, K. , Rottinghaus, G. E. , Chen, Y. C. , Kliethermes, B. , & Sauter, E. R. (2012). Trans‐resveratrol alters mammary promoter hypermethylation in women at increased risk for breast cancer. Nutrition and Cancer, 64(3), 393–400. 22332908 10.1080/01635581.2012.654926 PMC3392022

[fsn33933-bib-0162] Zupančič, Š. , Lavrič, Z. , & Kristl, J. (2015). Stability and solubility of trans‐resveratrol are strongly influenced by pH and temperature. European Journal of Pharmaceutics and Biopharmaceutics, 93, 196–204. 25864442 10.1016/j.ejpb.2015.04.002

